# Translocation of cytosolic human Cdc73 to stress granules plays a role in arsenic stress-induced stabilization of p53 mRNA

**DOI:** 10.1242/jcs.260593

**Published:** 2023-07-20

**Authors:** Hojin Lee, Tae-Hyeon Kim, Joo-Yeon Yoo

**Affiliations:** Department of Life Sciences, Pohang University of Science and Technology (POSTECH), Cheongam-Ro 77, Pohang, Geyongbuk 37673, South Korea

**Keywords:** Stress granule, PAFc, Cdc73, Parafibromin, p53 mRNA, Intrinsically disordered region

## Abstract

Cells trigger the assembly of stress granules (SGs) under various stress conditions. Among the many proteins recruited to SGs are RNA-binding proteins and transcription regulators. Here, we report the translocation of human (h)Cdc73, a component of the PAF1 transcription complex, to cytosolic SGs in response to arsenic stress. The hCdc73 protein possesses a long intrinsically disordered region (IDR) from amino acids 256–416, the presence of which is required for the translocation of hCdc73 to cytosolic SGs. The purified hCdc73 IDR formed droplets *in vitro*, and the light-activated assembly of hCdc73-IDR–mCherry–CRY2 was verified. For translocation of hCdc73 to SGs, physical interactions with SG carrier proteins, such as FMR1, are also needed. Previously, we reported that the cytosolic hCdc73–eEF1Bγ complex controls the stability of p53 mRNA. Under arsenic stress, selective sequestration of cytosolic hCdc73, but not eEF1Bγ (*EEF1G*) or p53 (*TP53*) mRNA, was detected. As a result, a transient increase in p53 mRNA at the post-transcriptional level was observed. In conclusion, we propose that the availability of mRNAs for stress-responsive genes can be controlled by restraining their negative regulators within SGs.

## Teaser

**Summary:** When exposed to harmful environmental conditions, cells trigger stress granule formation to reprioritize stress responsive genes (p53 mRNA) by sequestering its negative regulator hCdc73.

## INTRODUCTION

Organisms have evolved ways to respond to internal and environmental stresses for the maintenance of homeostasis and survival (Kultz, 2005; Galluzzi et al., 2018). The process, collectively known as the cellular stress response, is triggered by various hazardous conditions, including the presence of oxidants, genotoxins, metabolic inhibitors and extreme temperatures (Martindale and Holbrook, 2002; Ciccia and Elledge, 2010; Richter et al., 2010; Wang and Lei, 2018). In response to stress, cells activate a signaling cascade to suppress global levels of translation and to reorganize gene expression by selectively inducing the expression of a specific set of genes from a pool of presynthesized mRNAs (Pizzinga et al., 2020; Fan et al., 2002). This is an efficient and safe way to respond immediately to cellular stress signals without a significant time delay in new mRNA synthesis.

Under cellular stress conditions, mRNAs that are not undergoing translation and stalled mRNAs preferentially bind various RNA-binding proteins (RBPs) and are stored within nuclear bodies or cytoplasmic bodies, the assembly of which is controlled by liquid–liquid phase separation (LLPS) (Buchan, 2014; Shevtsov and Dundr, 2011; Banani et al., 2017). One of the most studied types of cytoplasmic bodies that function during the cellular stress response is the stress granule (SG). Nontranslating mRNAs and translation initiation components are found within SGs, suggesting the storage function of SGs during stress responses. SGs are expected to influence gene expression by directly modulating translation through the relocation of essential regulatory proteins for translation and mRNAs away from the protein synthesis machinery (Protter and Parker, 2016; Panas et al., 2016). The assembly of SGs is also directly induced by translational arrest, as the release of bulky polysome-free mRNAs triggers SG assembly along with stalled translation initiation complexes, which compartmentalize the mRNAs from cytosol, thereby protecting nontranslating mRNAs from degradation (Kimball et al., 2003).

Recent transcriptomic and proteomic studies of SGs have confirmed that stalled translation factors, in addition to RNAs and various RBPs, are major constituents of SGs (Youn et al., 2019). In contrast to the projected role of SGs in global mRNA protection, however, relatively small levels of mRNAs are actually enriched within SGs (Khong et al., 2017; Van Treeck et al., 2018). Furthermore, reporter mRNAs designed to localize to SGs are translated and degraded at rates similar to those of their cytosolic counterparts (Wilbertz et al., 2019; Mateju et al., 2020). Although stress-induced compartmentalization of ribonucleoprotein (RNP) granules provides an effective means of spatiotemporal regulation of gene expression, how RNP granules selectively orchestrate the expression of stress-responsive genes is not clear. Interestingly, regulators of transcription and nucleocytoplasmic transporters are also included among the proteome of SGs (Jain et al., 2016; Markmiller et al., 2018; Youn et al., 2018; Marmor-Kollet et al., 2020). As evidenced by the neurodegenerative behavior of nucleocytoplasmic transport factors sequestered in SGs (Zhang et al., 2018), the selective recruitment of transcription regulators into SGs suggests that they might function to specifically control the expression of stress-responsive genes. It has also been reported that transcription regulators with neurodegenerative mutations sequester themselves within SGs, which disrupts their nuclear localization and function (Vance et al., 2013).

Human (h)Cdc73 (also known as parafibromin) is one of the components of the human RNA polymerase II (RNAPII)-associated factor complex (PAFc). PAFc is a complex of Ctr9, Paf1, Leo1 and Rtf1, in addition to hCdc73. Ski8 (also known as SKIC8), a component of RNA exosomes, has also been found to be associated with PAFc in humans (Jaehning, 2010; Rozenblatt-Rosen et al., 2005; Zhu et al., 2005). Through protein–protein interactions, PAFc recruits transcription regulatory proteins and chromatin modifiers to target genomic loci and is thereby involved in almost every step of transcription mediated by RNAPII (Jaehning, 2010; Van Oss et al., 2017). A previous cryo-EM study of human PAFc has illustrated a loosely interconnected conformation of PAFc in which each component interacts with a different subunit of RNAPII. Although the Leo1-bound forms of Paf1 and Ctr9 sit on the outer boundary of RNAPII, the association of hCdc73 with PAFc or RNAPII is less stable, and hCdc73 even seems mobile (Vos et al., 2020).

In support of the above discovery, the PAFc-independent behavior of hCdc73 has been widely reported. hCdc73 acts as a nuclear platform/scaffold protein to integrate and convert signals conveyed by the Wnt, hedgehog or Notch morphogen pathways, depending on its phosphorylation status (Takahashi et al., 2011; Kikuchi et al., 2016). hCdc73 physically associates with the mRNA processing complex consisting of cleavage and polyadenylation specificity factor (CPSF) and cleavage stimulation factor (CstF), which is necessary for proper 3′ end modification of mRNAs in the nucleus (Rozenblatt-Rosen et al., 2009). hCdc73 also forms a stable complex with eukaryotic elongation factor 1B (eEF1Bγ, encoded by *EEF1G*) and Ski8 in the cytosol (Jo et al., 2014). Through its interaction with Ski8 and eEF1Bγ, which directly bind the mature form of p53 (*TP53*) mRNA, hCdc73 controls the stability of p53 mRNA and p53-mediated apoptosis (Jo et al., 2014). In addition to its canonical role in translation, the RNA-binding properties of eEF1Bγ allow it to play a pivotal role in transcription, especially in p53-mediated cellular stress responses (Pisani et al., 2016).

hCdc73 has been observed within the nuclear amyloid body, the protein assembly of which is induced by cellular stress signals (Marijan et al., 2019). Moreover, both human and yeast Cdc73 have also been identified among the proteomes of cytosolic SGs (Jain et al., 2016; Boncella et al., 2020). Here, we experimentally demonstrate that cytosolic hCdc73 translocates to SGs and that its translocation contributes to the spatiotemporal regulation of p53 mRNA under cellular stress conditions.

## RESULTS

### hCdc73 translocates to SGs under various cellular stress conditions

**Fig. 1.**
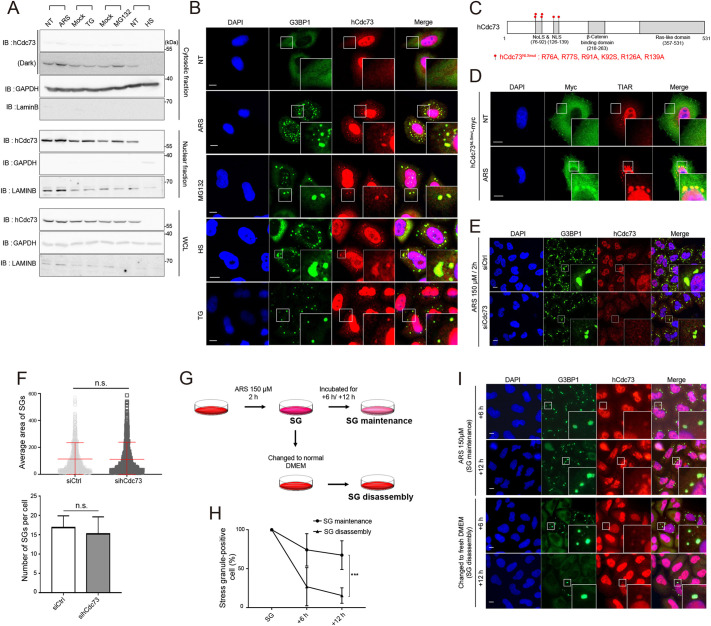
**hCdc73 translocates to SGs under various stress conditions.** (A) Immunoblot analysis (IB) of hCdc73 from the cytosol (top), nucleus (middle), and whole-cell lysates (WCL, bottom). HeLa cells were treated with 500 μM sodium arsenite for 1 h (ARS), 50 μM thapsigargin for 1 h (TG), 10 μM MG132 for 4 h (MG132) and 46°C heat shock for 1 h (HS). Each sample was loaded next to its own control set. NT, nontreated; Mock, DMSO treated. GAPDH and LAMINB were used as marker proteins for the cytosol and nuclear fractions, respectively. (B) Confocal images of endogenous hCdc73 and G3BP1 in cells either untreated (NT) or stressed as indicated. The same conditions in A were used except for the heat-shock condition, which was 43°C for 30 min. (C) Schematic diagram of the mutation sites used to generate hCdc73^NLSmut^. (D) Confocal images of hCdc73^NLSmut^–Myc-transfected HeLa cells that were untreated (NT) or treated with 500 μM sodium arsenite for 1 h (ARS). TIAR, marker for SGs. (E) Confocal images of endogenous hCdc73 and G3BP1 in HeLa cells transfected with siRNA against hCdc73 (siCdc73) or control diRNA (siCtrl) treated with 150 μM sodium arsenite for 2 h. (F) Area (top) and number of SGs per cell (bottom) quantified from the data in E (*n*=51). (G) Schematic diagram of the SG maintenance and disassembly experiment. (H) Percentage of SG-positive cells measured during SG maintenance and disassembly conditions (*n*=50–99). (I) Representative confocal images of endogenous hCdc73 and G3BP1 tested at an additional six and 12 h of SG maintenance and disassembly conditions. Scatter plot graphs and bar graphs are presented as the mean±s.d. ***P<0.0005; n.s., not significant (unpaired two-tailed *t*-test). All images and blot data are representative of at least three biologically independent experiments. Insets in B, D, E and I show a magnification of the indicated area. Scale bars: 10 μm. *P<0.05; **P<0.005; ***P<0.0005; ****P<0.00005; n.s., not significant.

In a previous study, we observed that hCdc73 in the cytosol functions to regulate the stability of mature p53 mRNA via its interaction with eEF1Bγ and Ski8 (Jo et al., 2014). Given that hCdc73 is normally located and functions in the nucleus as a component of the PAFc, we hypothesized that the cytosolic localization and related function of hCdc73 in the cytosol might be subject to control. To search for physiological conditions that affect the cytosolic localization of hCdc73, we tested various stress signals that induce the formation of SGs. Upon the treatment of cells with sodium arsenite, a representative reagent that causes oxidative stress and the unfolded protein response (UPR) (Bernstam and Nriagu, 2000), or MG132, an inhibitor of 26S proteasomal degradation (Rock et al., 1994), a slight increase in the amount of cytosolic hCdc73 was detected (Fig. 1A). At the same time, the transient assembly of cytosolic hCdc73 was observed under fluorescence microscopy, and cytosolic hCdc73 coincided with the typical marker proteins of SGs, G3BP1 and TIA-1-related protein (TIAR; also known as TIAL1) (Fig. 1B; Fig. S1A). In the case of thapsigargin (TG), which causes ER stress (Lytton et al., 1991), a dramatic change in cytosolic Cdc73 levels was not detected (Fig. 1A). On the other hand, upon heat shock stress, hCdc73 levels diminished in the cytosolic fraction and were observed in the insoluble fraction (Fig. S1B), suggesting that a major population of hCdc73 formed aggregates under these harsh conditions (Fig. S1C). Although the number and morphology of SGs differed, colocalization of hCdc73 with SGs was detected under every condition tested for SG induction (Fig. 1B; Fig. S1D). The translocation of cytosolic hCdc73 to SGs at various concentrations of sodium arsenite was separately verified (Fig. S1E). In addition to HeLa cells, we also confirmed the localization of hCdc73 within SGs in HEK-293 and U-2OS cells (Fig. S1F). Of note, although we consistently observed translocation of hCdc73 to SGs, this does not mean that all of the cytosolic hCdc73 protein pool translocates to SGs. A proportion of the cytosolic hCdc73 translocate to SGs, while a large pool of hCdc73 remains in the cytosol.

The localization of cytosolic hCdc73 within SGs was also tested with exogenously transfected hCdc73–Myc. Although colocalization of hCdc73–Myc with G3BP1 assembles was observed, the overall intensity of cytosolic hCdc73–Myc was weak (Fig. S1G). It seems that expression of the transfected hCdc73–Myc was overrepresented in the nucleus. To increase the amount of cytosolic hCdc73 protein, we generated mutant forms of hCdc73 in which its nuclear and nucleolar localization sequences were mutated (Fig. 1C). hCdc73 possesses a functional nuclear localization signal (NLS) from amino acids 126–139 and a nucleolar localization signal (NoLS) from amino acids 76–92 (Hahn and Marsh, 2005, 2007). As expected, the mutation of both NLS and NoLS (hCdc73^NLSmut^; R76A, R77S, R91A, K92S, R126A and R139A) resulted in the preferential expression of hCdc73 in the cytosol (Fig. 1D). hCdc73^NLSmut^–Myc-expressing cells were then stimulated with sodium arsenite, and the colocalization of hCdc73^NLSmut^–Myc proteins with the SG marker protein TIAR was observed. As observed with endogenous hCdc73, only a proportion of the cytosolic hCdc73^NLSmut^–Myc colocalized with SGs. Observing the colocalization patterns of hCdc73 and SGs, we wondered whether there is any possibility of hCdc73 controlling the SG assembly processes itself. However, when the expression of endogenous hCdc73 was silenced (Fig. S1H), the area and number of G3BP1-positive SGs were unaffected, indicating that hCdc73 is unlikely to function during SG formation processes (Fig. 1E,F).

The formation of SGs is a reversibly regulated process, as the assembly and disassembly of SGs are dynamically controlled. We therefore questioned what would happen to assembled hCdc73 when SGs were dissociated. To address this question, HeLa cells were incubated with 150 µM sodium arsenite for 2 h and then cultured in normal culture medium for an additional 6 or 12 h to remove stress inducers (Fig. 1G, ‘SG disassembly’) or were continuously maintained in sodium arsenite-containing medium (Fig. 1G, ‘SG maintenance’). During recovery periods in normal medium, the percentage of SG-containing cells dramatically declined, indicating that SGs had disassembled. After 12 h of recovery in normal medium, only 14% of cells possessed SGs (Fig. 1H; Fig. S1I). In contrast, when cells were continuously maintained in sodium arsenite-containing medium, as a control, sustained assembly of SGs (64%) was observed, even after 12 h of incubation (Fig. 1H). Under this harsh condition, ∼70% of cells were still viable, without significant changes in cellular morphology (Fig. S1I,J). Using these experimental schemes, we examined the localization of endogenous hCdc73 relative to SGs. In accordance with the disappearance of SGs, the punctuate form of cytosolic hCdc73 also disappeared. This disappearance was not due to a change in its cytosolic protein level (Fig. S1K). Given that assembled cytosolic hCdc73 was detected only when SGs were present (Fig. 1I), we conclude that the cytosolic hCdc73 protein is likely disassembled back into the cytosol.

### An IDR within the hCdc73 protein is required for its translocation to SGs

**Fig. 2.**
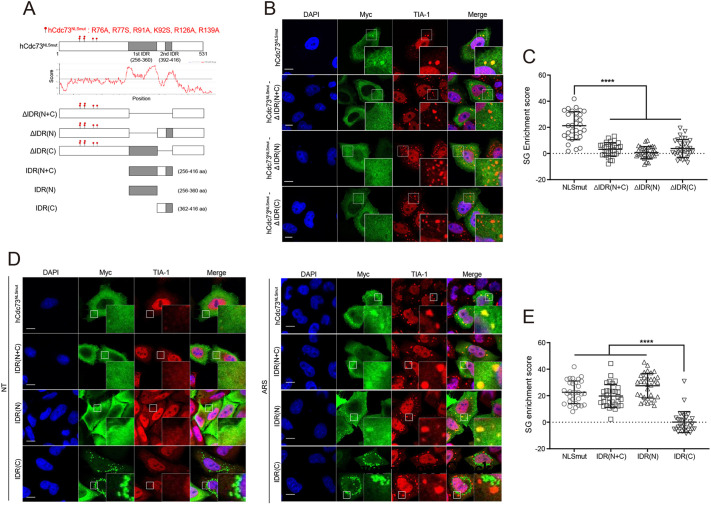
**An IDR within the hCdc73 protein is required for its translocation to SGs.** (A) Schematic diagram of various hCdc73 IDR mutants. Owing to the mutation sites within hCdc73^NLSmut^, ΔIDR plasmid constructs were cloned from the hCdc73^NLSmut^ plasmid as indicated. The presented IDR score of hCdc73 was obtained from the IUPred simulation program (Dosztãnyi et al., 2005). The disordered region is indicated by a gray box. (B) Representative confocal images of HeLa cells transfected with hCdc73^NLSmut^–Myc and IDR deletion mutants. The images were taken after treatment with 500 μM sodium arsenite for 1 h. TIA-I is a marker for SGs. (C) Quantification of SG enrichment score for hCdc73^NLSmut^–Myc (NLSmut) and IDR deletion mutants within SGs (*n*=30). (D) Representative confocal images of HeLa cells transfected with hCdc73^NLSmut^-Myc and the truncated IDR form of hCdc73. Cells were untreated (NT, left) or treated with 500 μM sodium arsenite for 1 h (ARS, right). (E) Quantification of SG enrichment score for hCdc73^NLSmut^–Myc and the truncated IDR form of hCdc73 within SGs (*n*=30). The SG enrichment score of each hCdc73 IDR mutant was measured as described in the Materials and Methods section. Scatter plot graphs are presented as the mean±s.d. ****P<0.00005 (one-way ANOVA with multiple comparison test). All images are representative of at least three biologically independent experiments. Insets in B and D show a magnification of the indicated area. Scale bars: 10 μm.

To understand how hCdc73 translocates to or assembles within SGs, we attempted to identify the domains responsible for hCdc73 that control this behavior. hCdc73 contains a C-terminal Ras-like domain that has homology with yeast Cdc73, and known interaction domains for β-catenin or for eEF1Bγ and Ski8 lie within the N-terminal region (Jo et al., 2014; Amrich et al., 2012; Mosimann et al., 2006). In addition, most cancer-related mutations are observed within the N-terminal region of hCdc73, indicating the functional importance of this region (Newey et al., 2010). Although the crystal structures of the C-terminal domain of yeast Cdc73 and the N-terminal half (amino acids 1–111) of hCdc73 have been separately reported (Amrich et al., 2012; Sun et al., 2017), the crystal structure of the full-length form of hCdc73 is not available, suggesting that unstructured low-complexity regions [i.e. intrinsically disordered region (IDRs)] might be present within hCdc73. To address this hypothesis, we utilized the D^2^P^2^ program (Oates et al., 2013) to search for low-complexity regions within hCdc73 (Fig. S2A,B). Broad regions encompassing amino acids 256–416 scored high for low complexity; of these residues, the region could be further subdivided into ‘IDR(N)’, which covers the first N-terminal half of the IDR (amino acids 256–360), and ‘IDR(C)’, covering the second IDR domain (amino acids 361–416) (Fig. 2A). As the LLPS properties of intrinsically disordered proteins are one of the main factors for the assembly of SGs, we focused on these putative IDR domains of hCdc73.

Thus, hCdc73 mutants that lack the individual IDR(N) or IDR(C) region, or both, were generated and tested for their capability to translocate into SGs (Fig. 2A,B). This time, we used TIA-1 as an SG protein marker, which is a known scaffold protein that mediates SG formation (Gilks et al., 2004). Unlike the full-length form of hCdc73, all of the hCdc73 deletion mutants tested failed to translocate to SGs. To quantify the colocalization of hCdc73 with SGs, we calculated the SG enrichment score, which measures the fluorescence intensity of hCdc73 within SGs (Fig. 2C). These data suggested that both IDR(N) and IDR(C) regions are needed for hCdc73 to translocate to SGs.

To support this idea, we next generated a truncated form of hCdc73, IDR(N+C), and tested its translocation to SGs (Fig. 2A,D). Without stress, the IDR(N+C) protein signal was dispersed in the cytosol. Under arsenic stress conditions, most of the IDR(N+C) protein signal remained in the cytosol, but small amounts of IDR(N+C) proteins were found where SGs formed, indicating that this fragment of protein was able to translocate to SGs. As a control, we also tested IDR(N) and IDR(C) (Fig. 2D,E). To our surprise, portions of IDR(N) were found where SGs formed, whereas IDR(C) was found outside SGs in an aggregated form. In fact, IDR(C) forms aggregates, even in the absence of arsenic stress, indicating that there are strong interactions between IDR(C) and other IDR(C) molecules.

### The hCdc73 IDR possesses phase separation properties

**Fig. 3.**
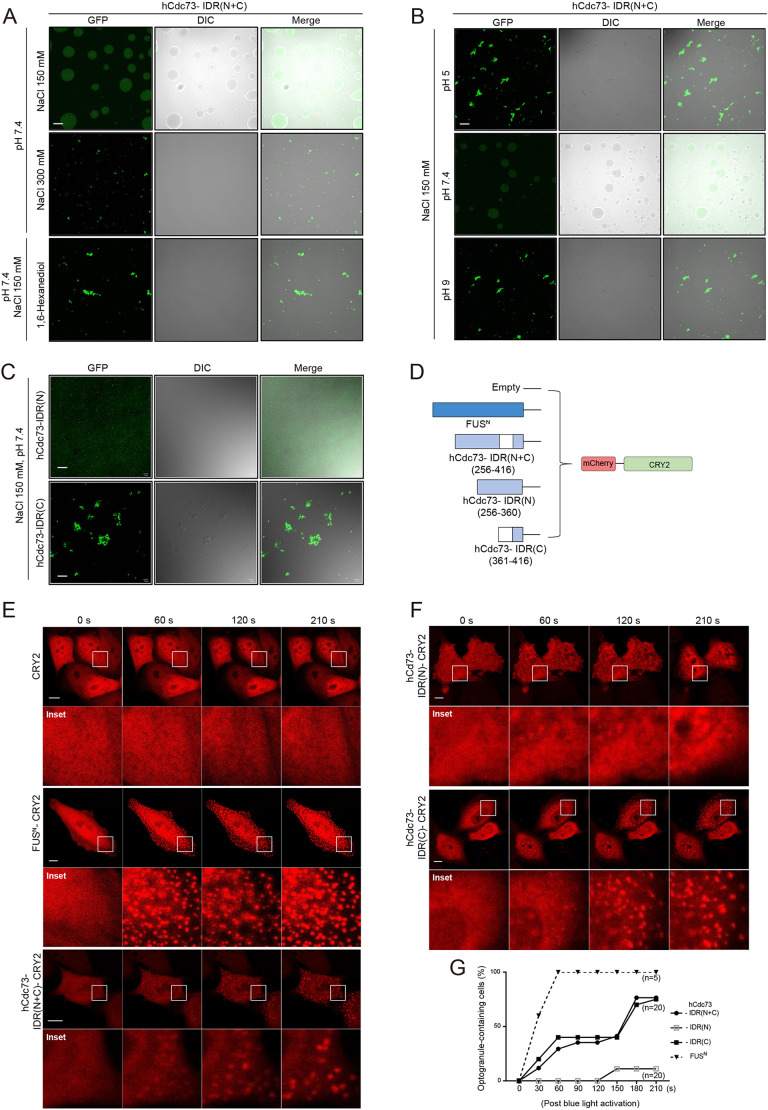
**hCdc73-IDR possesses phase separation properties.** (A–C) Representative images of the *in vitro* droplet formation assay. (A,B) Recombinant hCdc73-IDR(N+C)–GFP (10 μM) was incubated at the indicated pH and salt concentration with 10% PEG8000. 1,6-Hexanediol (5%) was applied, where indicated, to disturb weak hydrophobic interactions. DIC, differential interference contrast view. (C) Recombinant hCc73-IDR(N)–GFP and hCc73-IDR(C)–GFP (10 μM) were incubated at the indicated pH and salt concentration with 10% PEG8000. (D) Schematic diagram of the constructed optoDroplet plasmids. As a positive control, the IDR of FUS (FUS^N^) was used. (E,F) Time-lapse imaging of droplet formation upon blue light exposure in HeLa cells. (G) Quantification of optoDroplet-containing cells; data from E and F, *n*=20, except FUS^N^ (*n*=5). All images are representative of at least three biologically independent experiments. Insets in E and F show a magnification of the indicated area. Scale bars: 10 μm.

Proteins with intrinsically disordered regions can undergo LLPS to form membraneless organelles. In addition to the scaffolding proteins of SGs, such as G3BP1 and G3BP2, many other proteins that assemble within SGs are intrinsically disordered proteins (IDPs) or tend to possess IDRs (Youn et al., 2019; Nott et al., 2015; Molliex et al., 2015). Therefore, we tested whether the IDR of hCdc73 also guarantees the ability to undergo phase separation and whether this property is needed for the translocation of hCdc73 to SGs. First, we tried to purify the full-length form of the hCdc73 protein *in vitro* but failed. Therefore, we next attempted to purify the IDR(N+C) domain of hCdc73. Recombinant 6xHis-GFP-tagged IDR(N+C) of hCdc73 was successfully purified and tested for its ability to form droplets *in vitro* (Fig. S2C). With the aid of a crowding reagent [10% polyethylene glycol (PEG) 8000], the purified protein at a 10 μM concentration formed liquid droplets under physiological buffer conditions (150 mM NaCl, pH 7.4) (Fig. 3A). Assembly into liquid droplets was sensitive to the salt concentration, as liquid droplets were barely detected under high-salt (300 mM NaCl) conditions. Acidic or basic buffer conditions also similarly prevented droplet formation events (Fig. 3B). Under these pH conditions, hCdc73-IDR(N+C) seemed to aggregate. When the purified hCdc73-IDR(N+C) protein was treated with 1,6-hexanediol at pH 7, the droplets disappeared, indicating that weak hydrophobic interactions are important for this assembly event (Fig. 3A). Of note, the aggregates observed at pH 5 and pH 9 were resistant to 1,6-hexanediol treatment. In contrast to what was seen with hCdc73-IDR(N+C), neither purified hCdc73-IDR(N) nor hCdc73-IDR(C) formed liquid droplets *in vitro* (Fig. 3C). Similar to the aggregated expression patterns observed in transfected cells (Fig. 2D), purified IDR(C) protein also aggregated *in vitro*. These results indicate that IDR(C)–IDR(C) molecular interactions might be too strong to undergo LLPS, whereas the presence of IDR(N) somewhat loosens the interaction strength for IDR(C)–IDR(C), such that it is low enough to undergo phase separation.

To further test the phase-separating property of the IDR, we performed optoDroplet assays in transfected cells (Shin et al., 2017). For this, hCdc73-IDR(N+C)–mCherry was fused to CRY2, a light-sensitive self-associating protein, and the light-inducible phase separation properties of hCdc73-IDR were examined after blue light exposure (Fig. 3D). As a positive control, the disordered N-terminal domain of FUS (FUS^N^; amino acids 1–214), which covers the prion-like domain, was included. Upon blue light (488-nm laser)-induced activation, hCdc73-IDR(N+C)–mCherry–CRY2 underwent light-inducible phase separation, resulting in distinct droplet assembly (Fig. 3E). In contrast to FUS^N^, however, the kinetics of droplet assembly were rather slow. Under our experimental conditions, the phase separation of FUS^N^–mCherry–CRY2 was detected after 30 s of exposure, and most of the tested cells formed droplets after 60 s of light exposure (Fig. 3E,G). However, light-induced droplets of hCdc73-IDR(N+C)–mCherry–CRY2 were barely detected after 30 s of exposure and only started to appear after 60 s of exposure. Even after longer light exposure, not every cell tested formed droplets (Fig. 3E,G), indicating that although hCdc73-IDR(N+C) is able to undergo phase separation, it possesses relatively weak activity. As a control, we also tested whether the full-length form of hCdc73^NLSmu^–mCherry–CRY2 undergoes phase separation. However, as easily expected from the relatively weak potency of Cdc73-IDR, light-induced assembly of hCdc73^NLSmut^–mCherry–CRY2 was not detected (Fig. S2D). Finally, we tested hCdc73-IDR(N)–mCherry–CRY2 and hCdc73-IDR(C)–mCherry–CRY2 for their potential to assemble by itself (Fig. 3F). In support of the *in vitro* droplet assay results, hCdc73-IDR(N)–mCherry–CRY2 did not form any kinds of droplets under the conditions we tested. In contrast, hCdc73-IDR(C)–mCherry–CRY2 exhibited phase separation properties similar to those observed with hCdc73-IDR(N+C)–mCherry–CRY2 (Fig. 3F,G). This was somewhat surprising, as the aggregated form of IDR(C) was detected *in vitro* but not in the transfected cells (Fig. 3C,F). We postulate that the large-sized fusion protein mCherry2–CRY2 at the C-terminal end might interfere with the strong IDR(C)–IDR(C) interactions.

### The carrier protein FMR1 supports the translocation of hCdc73 to SGs

**Fig. 4.**
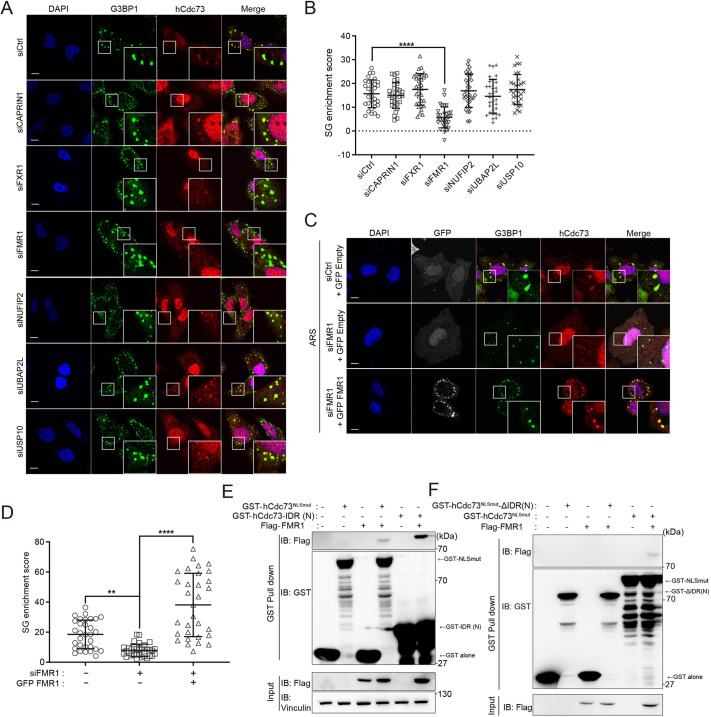
**The carrier protein FMR1 supports the translocation of hCdc73 to SGs.** (A) Representative confocal images of endogenous hCdc73 and G3BP1 in siRNA-transfected HeLa cells. The cells were transfected with the indicated siRNAs followed by 150 μM sodium arsenite treatment for 2 h. (B) SG enrichment scores obtained from the data in A (*n*=30). (C) Representative confocal images of endogenous hCdc73, G3BP1 and exogenous GFP or GFP-tagged FMR1 protein. HeLa cells were transfected with siRNA as indicated and then reconstituted with GFP-Empty or GFP-FMR1, followed by arsenic stress conditions (ARS, sodium arsenite 150 μM for 2 h). (D) SG enrichment scores obtained from the data in C (*n*=30). (E,F) HEK-293T cells were transfected with the indicated plasmids and subjected to a GST pulldown assay. ‘–’ indicates GST-empty or Flag-empty vector, respectively. All scatter plot graphs are presented as the mean±s.d. **P<0.005; ****P<0.00005 (one-way ANOVA with multiple comparison test). All images are representative of at least three biologically independent experiments. Insets in A and B show a magnification of the indicated area. Scale bars: 10 μm.

SGs are composed of ‘core’ or ‘scaffold’ proteins surrounded by less-concentrated ‘shell’ or ‘client’ proteins, along with various mRNAs (Jain et al., 2016; Banani et al., 2016). Given that the phase separation properties of hCdc73 IDRs are necessary but not sufficient for the recruitment of hCdc73 within SGs, we speculated that hCdc73 likely requires specific protein–protein interactions with scaffolding proteins. To address this possibility, we first tested whether hCdc73 physically interacts with G3BP1, a major core protein of SGs. However, we did not observe specific physical interactions between these proteins (Fig. S3A,B). Therefore, we next asked whether the recruitment of hCdc73 to SGs depends on physical interactions with carrier proteins, which are heavily associated with SGs. In our search for putative carrier proteins associated with SGs, we first selected six candidate proteins that are abundant and frequently studied (Sanders et al., 2020). HeLa cells were separately transfected with siRNAs targeting each candidate protein, and the colocalization of cytosolic hCdc73 to sodium arsenite-induced SGs was examined (Fig. 4A,B; Fig. S3C). Among the tested proteins, the silencing of FMR1 showed the most dramatic effect, as the recruitment of cytosolic hCdc73 to sites where G3BP1 assembles was significantly decreased (Fig. 4B). Although gene expression was repressed accordingly, the silencing of CAPRIN1, FXR1, NUFIP2, UBAP2L or USP10 did not dramatically change the colocalization patterns of hCdc73 and SGs. The importance of FMR1-mediated recruitment of hCdc73 to SGs was separately confirmed by GFP–FMR1 rescue experiments in FMR1-silenced cells (Fig. 4C,D). To avoid possible degradation of plasmid-derived FMR1 by siRNA, we used siRNAs targeting the 3′-UTR of the FMR1 gene (Table S1).

Given that the proteomic database of the FMR1 interactome includes hCdc73 (Taha et al., 2021), we hypothesized that FMR1 functions to aid, probably via physical interactions, the localization of hCdc73 to SGs. To address whether FMR1 indeed physically interacts with hCdc73 in our experimental setting, a GST pulldown assay using the lysates of Flag–FMR1- and GST–hCdc73^NLSmut^-transfected HeLa cells was performed. As expected, GST–hCdc73^NLSmut^ interacted with the full-length form of the FMR1 protein (Fig. 4E). Moreover, GST–hCdc73-IDR(N) was able to interact with FMR1. In contrast, when GST–hCdc73^NLSmut^-ΔIDR(N) was tested, interaction with FMR1 was not detected, suggesting that hCdc73 likely interacts with FMR1 through its IDR(N) domain (Fig. 4F). In addition to FMR1, hCdc73 also interacted with CAPRIN1, a known binding partner of FMR1 (El Fatimy et al., 2012), but not with USP10 (Fig. S3D). Our data collectively suggest that hCdc73 physically interacts with FMR1, which mediates its translocation into SGs.

### Levels of p53 mRNA and SG assembly are linked under arsenic stress conditions

**Fig. 5.**
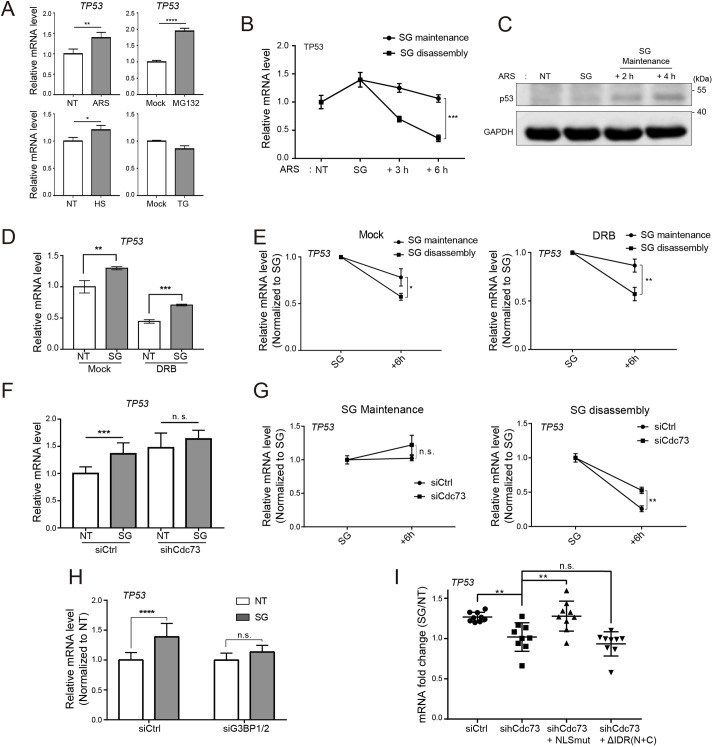
**Translocation of hCdc73 to SGs in part controls cytosolic p53 mRNA pools.** (A) HeLa cells were treated with 500 μM sodium arsenite for 1 h (ARS), 10 μM MG132 for 4 h, 50 μM thapsigargin for 1 h (TG) and heat shock at 46°C for 1 h (HS). The p53 mRNA level in each stressed cell type was measured by quantitative (q)RT-PCR and compared with its own control set (*n*=3). NT, nontreated; Mock, DMSO-treated. (B) HeLa cells were treated with 250 μM sodium arsenite (ARS) for 1 h (SG), followed by SG maintenance or disassembly conditions (for 3 h or 6 h). At the indicated points, p53 mRNA levels were measured by qRT-PCR (*n*=3). (C) Immunoblot analysis of p53 from the whole-cell lysate. HeLa cells were treated with 150 μM sodium arsenite for 2 h (SG) and an additional 2 or 4 h. (D,E) HEK-293 cells were treated with DRB and sodium arsenite as indicated in Fig. 4G, 150 μM of sodium arsenite was used for 2 h (SG), followed by SG maintenance or disassembly conditions for an additional 6 h. p53 mRNA levels were measured by qRT-PCR (*n*=3). (F) Control (siCtrl) or hCdc73 siRNA (siCdc73)-transfected HeLa cells were treated with 150 μM sodium arsenite for 2 h (SG) (*n*=9). p53 mRNA (*TP53*) levels were measured. (G) siRNA-transfected HeLa cells were treated with 150 μM sodium arsenite (SG) followed by SG maintenance or disassembly conditions for the indicated times (*n*=3). p53 mRNA levels in F and G were measured by qRT-PCR. (H) Control or G3BP1/2 siRNA-transfected HeLa cells were treated with sodium arsenite (150 μM, 2 h; SG). p53 mRNA levels were measured by qRT-PCR (*n*=9). (I) hCdc73 siRNA-transfected HEK-293 cells were reconstituted with hCdc73^NLSmut^–Myc (NLSmut) or hCdc73^NLSmut^-ΔIDR(N+C)–Myc. After arsenic stress (150 μM, 2 h), the fold change in p53 mRNA levels (SG compared to NT) was measured by qRT-PCR (*n*=9). **P<0.005; n.s., not significant (one-way ANOVA with multiple comparison test). hRPL32 was used as the internal control in all the qRT-PCR data above. Each graph represents the mean±s.d. data from three independent experiments. *P<0.05; **P<0.005; ***P<0.0005; ****P<0.00005; n.s., not significant (unpaired two-tailed *t*-test except for I).

SG-inducing cellular stressors trigger global translational arrest that results in a high concentration of nontranslating RNAs in the cytosol (Protter and Parker, 2016; Panas et al., 2016). The compartmentalization of RNAs within SGs is predicted to provide a protective shelter for ribosome-free RNAs. However, a recent study has also suggested that SG formation is an adaptive response that selectively renders stress-responsive mRNAs available (Riback et al., 2017). Given that we previously observed that cytosolic hCdc73 controls the stability of p53 mRNA, we wondered whether the abundance of p53 mRNA is affected during SG assembly and, if so, whether hCdc73 plays any role in this regulation. To test this idea, we first examined whether p53 mRNA levels were altered under stress conditions that induce the assembly of SGs. Acute treatment with sodium arsenite or MG132 increased p53 mRNA levels up to 1.5-fold compared to those in mock-treated controls (Fig. 5A). Acute heat shock stress also increased p53 mRNA levels, but the effect was marginal. In the case of thapsigargin (TG), however, we did not observe changes in p53 mRNA levels. These data indicate that regulation of the p53 mRNA pool by SGs might not be a general event but occurs under specific stress situations. Given that we observed significant changes in p53 mRNA levels under arsenic stress-induced SG assembly conditions, we decided to address the role of cytosolic hCdc73 in the regulation of p53 mRNA pools under arsenic stress-induced SG conditions.

The effect of sodium arsenite at different doses on p53 mRNA levels was separately verified in other cell types, including HeLa, HEK-293 and U-2OS cells (Fig. S4A,B). To confirm whether the assembly of SGs and changes in p53 mRNA levels correlate, we next examined p53 mRNA levels during SG maintenance or disassembly conditions. As cell viability decreased after extended treatment with sodium arsenite (Fig. S1H,I), we maintained cells under sodium arsenite-induced stress conditions for an additional 3 h and 6 h after 1 h of acute treatment. In this case, we observed that the enhanced p53 mRNA level after acute treatment was sustained for an additional 6 h (Fig. 5B; Fig. S4C), in accordance with the proportion of SG-forming cells (Fig. 1H). In contrast, when arsenic-stressed cells were changed into normal medium for disassembly of SGs, p53 mRNA levels declined (Fig. 5B; Fig. S4C). Increased p53 mRNA levels reflected the protein levels, as under sustained SG-inducing conditions, the protein levels of p53 also increased (Fig. 5C). Similar patterns of p53 mRNA changes were also detected in MG132-treated cells (Fig. S4D–F).

To address whether the changes in p53 mRNA levels reflect controls at the transcriptional level or posttranscriptional level, cells were pretreated with 5,6-dichlorobenzimidazole 1-β-D-ribofuranoside (DRB), an inhibitor of transcription (Chodosh et al., 1989), and the formation of SGs and levels of p53 mRNA were examined. We pretreated cells with 100 µM DRB for 12 h, at which point p53 mRNA was halved (Fig. S4G,H). Notably, the amount of sodium arsenite-induced SG after pretreatment with DRB (100 µM) was not altered (Fig. S4I). Upon arsenic stress, p53 mRNA levels were increased regardless of DRB treatment (Fig. 5D). Additionally, similar patterns of p53 mRNA behavior under SG maintenance and disassembly conditions were observed, with or without DRB pretreatment (Fig. 5E). These data together indicate that changes in p53 mRNA levels under sodium arsenite-induced stress conditions are regulated at the post-transcriptional level.

### Translocation of hCdc73 to SGs in part controls cytosolic p53 mRNA pools

We next questioned the involvement of hCdc73 in the control of p53 mRNA levels under arsenic stress conditions. To address this question, p53 mRNA levels were examined in arsenic-stressed, hCdc73-silenced cells. As reported, hCdc73 silencing alone resulted in enhanced p53 mRNA levels (Jo et al., 2014). Upon arsenic stress, however, further induction of p53 mRNA was not detected in hCdc73-deficient cells (Fig. 5F), suggesting that arsenic stress might counterbalance the negative effect of hCdc73 on p53 mRNA. Separately, the effect of hCdc73 on p53 mRNA levels was also tested under SG maintenance and disassembly conditions (Fig. 5G). Under SG maintenance conditions, the changes in p53 mRNA levels were not significant, regardless of whether hCdc73 was depleted. By contrast, the decline in the p53 mRNA level during SG disassembly was substantially delayed in the hCdc73-depleted cells, indicating that the degradation of p53 mRNA during the recovery phases requires the presence of hCdc73.

It is possible that increased p53 mRNA levels resulted from the secondary effect of arsenic stress. To exclude this concern, we tested whether SG formation itself is important for changes in p53 mRNA levels. G3BP1 and G3BP2 are key scaffold proteins for SG formation (Kedersha et al., 2016) (Fig. S4J,K). Therefore, we evaluated p53 mRNA levels in arsenic-stressed cells silenced for both of these proteins (G3BP1/2-silenced cells). In G3BP1/2-silenced cells, arsenic stress could not enhance p53 mRNA levels (Fig. 5H), implying that the presence of SG is critical for p53 mRNA regulation. We further questioned whether translocation of hCdc73 to SGs is required for this event. To test this idea, hCdc73-silenced cells were reconstituted with hCdc73^NLSmut^–Myc or hCdc73^NLSmut^-ΔIDR(N+C)–Myc. Although both constructs were functionally active in controlling p53 mRNA stability and interaction with eEF1Bγ under normal conditions (Fig. S4L,M), their activity differed under SG-inducing conditions. Reconstituting cells with hCdc73^NLSmut^–Myc restored p53 mRNA regulatory activity under arsenic stress conditions (Fig. 5I). However, there was no detectable restoration in cells reconstituted with hCdc73^NLSmut^-ΔIDR(N+C)–Myc. Based on these data, we conclude that, under arsenic stress conditions, p53 mRNA levels are in part controlled by the translocation of hCdc73 to SGs.

### hCdc73, but not p53 mRNA or eEF1Bγ, translocates to arsenic stress-induced SGs

**Fig. 6.**
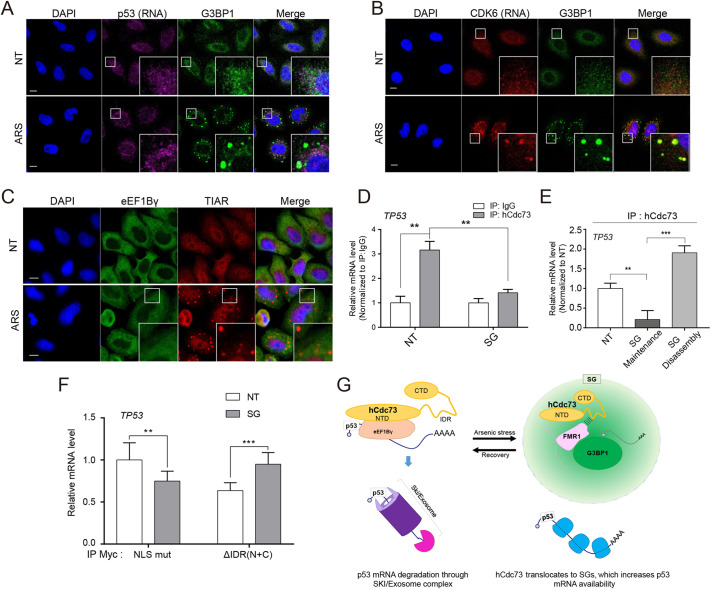
**hCdc73, but not p53 mRNA or eEF1Bγ, translocates to arsenic stress-induced SGs.** (A,B) Confocal images from RNA fluorescence *in situ* hybridization (RNA FISH) experiments. HeLa cells were either untreated (NT) or treated with 500 μM sodium arsenite for 1 h (ARS). FISH probes that specifically bind p53 (A) and Cdk6 (B) mRNAs were used. G3BP1 was used as a marker for SGs. (C) Confocal images of endogenous eEF1Bγ or TIAR in HeLa cells, either untreated (NT) or treated with 500 μM sodium arsenite for 1 h (ARS). (D) RNA immunoprecipitation (RNA-IP) was performed using IgG or endogenous hCdc73 antibodies and the cytosolic fraction of HEK-293 cells, either untreated (NT) or treated with 500 μM sodium arsenite for 1 h (ARS). Bound mRNA was measured by qRT-PCR (*n*=3). (E) Results of RNA-IP using endogenous hCdc73 antibody. HeLa cells were treated with 500 μM sodium arsenite for 1 h and then incubated for 6 h under SG maintenance or disassembly conditions (NT, untreated) (*n*=3). Bound mRNA was measured by qRT-PCR. (F) RNA-IP using Myc antibody. HeLa cells were transfected with hCdc73^NLSmut^–Myc (NLSmut) or hCdc73^NLSmut^-ΔIDR(N+C)–Myc and then treated with 500 μM sodium arsenite for 1 h (SG). NT, untreated. Bound mRNA was measured by qRT-PCR (*n*=9). hRPL32 was used as the internal control in all the qRT-PCR data above. Each graph represents the mean±s.d. from three independent experiments. **P<0.005; ***P<0.0005 (unpaired two-tailed *t*-test). All images are representative of at least three biologically independent experiments. Insets in A–C show a magnification of the indicated area. Scale bars: 10 μm. (G) Model of of hCdc73- and SG-mediated p53 mRNA control.

Our data thus far have demonstrated that (1) p53 mRNA levels increase at the post-transcriptional level when SGs assemble, (2) hCdc73 is required for this mRNA regulation and (3) hCdc73 is sequestered to SGs. Given that the cytosolic hCdc73–eEF1Bγ–Ski8 complex associates with p53 mRNA for degradation control (Jo et al., 2014), these observations led us to hypothesize that the entrapment of hCdc73 within SGs might work to prevent p53 mRNA degradation and to increase the cytosolic pool of p53 mRNA. To understand how this regulation occurs, we examined the hCdc73–eEF1Bγ–Ski8 complex and p53 mRNA during SG assembly. To do so, we first performed a fluorescence *in situ* hybridization (FISH) assay to visualize the spatial distribution patterns of p53 mRNA under arsenic stress conditions. A p53-specific RNA probe detected a broad p53 mRNA distribution in untreated cells. Upon sodium arsenite treatment, the spatial distribution pattern of p53 mRNA was not changed, and hence colocalization with G3BP1-positive SGs was not detected (Fig. 6A; Fig. S5A). Among the mRNAs reported to translocate to SGs (Khong et al., 2017; Van Treeck et al., 2018), we selected CDK6 mRNA as a positive control for FISH experiments. As expected, CDK6 mRNA colocalized well with G3BP1-positive SGs upon sodium arsenite treatment (Fig. 6B; Fig. S5B). Therefore, these data suggest that p53 mRNA is not included among the many other mRNAs stored within SGs during stress. A search for SG transcriptome data also revealed that p53 mRNA was not enriched in SGs (Khong et al., 2017; Van Treeck et al., 2018).

Next, we examined the spatial distribution patterns of the eEF1Bγ protein under arsenic stress conditions. eEF1Bγ was of particular interest, as the association of hCdc73 with p53 mRNA depends on this protein (Jo et al., 2014). Unlike hCdc73, however, recruitment of the eEF1Bγ protein into SGs was not detected through co-staining with the SG marker protein TIAR (Fig. 6C), and hence colocalization of eEF1Bγ along with hCdc73 was not detected at places where cytosolic hCdc73 assembled (Fig. S5C). In this condition, we also tested the physical interaction between hCdc73 and eEF1Bγ. Under basal conditions, a physical interaction between hCdc73 and eEF1Bγ was observed, which was slightly reduced under SG assembly conditions (Fig. S5D). Considering that fractions of cytosolic hCdc73 translocate to SGs, it is likely that sequestration of hCdc73 to SGs reduces the possibility of the hCdc73–eEF1Bγ–p53 mRNA complex forming under arsenic stress conditions. To confirm this hypothesis, we performed an RNA immunoprecipitation (RNA-IP) assay to detect associations between cytosolic hCdc73 and p53 mRNA (Fig. 6D; Fig. S5E). The data clearly demonstrated that the interaction between hCdc73 and p53 mRNA was significantly decreased under sodium arsenite-induced SG assembly conditions. When tested in SG disassembly conditions, the interaction between hCdc73 and p53 mRNA increased, again confirming that the entrapment of hCdc73 within SGs works to prevent the association of hCdc73 with p53 mRNA (Fig. 6E). Finally, we verified whether the translocation of hCdc73 to SGs through its IDR(N+C) lessens the interaction between hCdc73 and p53 mRNA. For this, we performed an RNA-IP assay with hCdc73^NLSmut^–Myc and hCdc73^NLSmut^-ΔIDR(N+C)–Myc, which failed to translocate to SGs (Fig. 2B). Unlike hCdc73^NLSmut^, arsenic stress-induced dissociation from p53 mRNA was not observed for hCdc73^NLSmut^-ΔIDR(N+C)–Myc (Fig. 6F). Based on these data, we conclude that the selective sequestration of hCdc73 away from the cytoplasmic RNP complex containing p53 mRNA functions to transiently increase the stability of p53 mRNA under cellular stress conditions (Fig. 6G).

## DISCUSSION

The tumor suppressor p53 is a transcription factor involved in the cellular decision of death and survival. It functions to inhibit cell proliferation and promote cell death when the cell is faced with detrimental cellular stress. Additionally, the activity of p53 is required for the maintenance of metabolic pathways that contribute to cell survival (Kruiswijk et al., 2015). For this reason, the abundance and activity of the p53 protein are tightly controlled during diverse stress conditions (Kruse and Gu, 2009). In addition to p53 production, the stability of p53 mRNA and protein are subject to control for more acute responses. Previously, we reported that hCdc73 forms a novel complex with eEF1Bγ and Ski8 that functions to degrade p53 mRNA in the cytosol (Jo et al., 2014). In the present report, we also demonstrate that p53 mRNA levels can be post-transcriptionally controlled under cellular stress conditions by sequestrating cytosolic hCdc73 into SGs (Fig. 6G).

Here, we observed that the levels of p53 mRNA correlated with SG assembly, especially when stressed with sodium arsenite. These changes in p53 mRNA levels during SG formation were independent of transcriptional events, as similar changes in p53 mRNA levels were detected when DRB, an inhibitor of transcription, was applied (Fig. 5E,F). Changes in p53 mRNA levels under arsenic stress conditions were sensitive to the assembly of SGs and translocation of hCdc73 to SGs. We observed that unlike many other mRNAs that are stored within SGs during arsenic stress, p53 mRNA remained in the cytosol (Fig. 6A). Under these conditions, the physical association of hCdc73 with p53 mRNA was disrupted (Fig. 6D). By contrast, when hCdc73 failed to translocate to SGs, the interaction between p53 mRNA and hCdc73 recovered (Fig. 6E,F). Based on these observations, we propose that the selective sequestration of hCdc73 into SGs functions to control cytosolic p53 mRNA pools under arsenic stress conditions.

For hCdc73 to be properly incorporated into SGs, the IDR(N+C) regions of hCdc73 are needed. Given that purified IDR(N+C) protein undergoes LLPS, we postulated that the IDR(N+C) domain of hCdc73 provides interphase interactions for assembly within SGs. In addition, IDR(N) serves as a key domain for physical interaction with the carrier protein FMR1. Quite interestingly, the interaction domain of hCdc73 for eEF1Bγ and FMR1 partially overlaps. In a previous report demonstrating the interaction between hCdc73 and eEF1Bγ, we showed that amino acids (aa) 110–343 of hCdc73 is required for its interaction with eEF1Bγ (Jo et al., 2014). In the present paper, we also demonstrate that the IDR(N) region (256–360 aa) of hCdc73 is needed for its interaction with FMR1. Given that those regions for interaction with eEF1Bγ overlap with those of FMR1, it is highly likely that their interaction with hCdc73 is competitive. Here, we demonstrate that FMR1, but not eEF1Bγ, can condense into SGs under arsenic stress conditions. Under basal conditions, the FMR1 and eEF1Bγ proteins are localized throughout the cytoplasm; hence, we speculate that the probability of dispersed hCdc73 protein encountering either FMR1 or eEF1Bγ in the cytosol is similar. In contrast, under stress conditions, cytosolic hCdc73 bound to FMR1 is selectively sequestered within SGs; hence, the probability of hCdc73 encountering eEF1Bγ outside SGs decreases. Therefore, in a scenario of random association of hCdc73 with competitive eEF1Bγ or FMR1, sequestration of hCdc73 along with FMR1 into restricted cellular compartments results in a lowered probability of eEF1Bγ (and p53 mRNA) association with hCdc73, which we observed.

From yeast to mammals, it is essential to reprioritize genes for transcription, from housekeeping genes to stress-responsive genes, upon exposure to harmful environmental conditions. Similar to what is seen for p53 mRNA control by the selective sequestration of hCdc73 within SGs during cellular stress conditions, Pab1-mediated heat shock-responsive gene regulation depends on the phase separation properties of Pab1. In yeast, the mRNAs of heat shock-responsive genes are bound by Pab1, which inhibits the translation of heat shock-responsive genes under normal cellular conditions. Under conditions of heat shock stress, Pab1 forms a biomolecular condensate, which frees the mRNAs of heat shock-responsive genes for translation (Riback et al., 2017). Unlike Pab1, which directly binds mRNA, the physical association of hCdc73 with p53 mRNA requires the RBP eEF1Bγ. However, the cytosolic distribution pattern of eEF1Bγ did not change upon SG formation, and eEF1Bγ remained in the cytosol along with p53 mRNA (Fig. 6C). In addition, the physical interaction between hCdc73 and eEF1Bγ was reduced upon sodium arsenite treatment (Fig. S5D). Although the detailed mechanisms differ, it seems that a mechanistically conserved stress response controls the availability of mRNA via selective sequestration of mRNA-associated cytosolic proteins into phase-separating granules.

Our data collectively show that p53 mRNA levels are post-transcriptionally regulated during cellular stress. Although p53 is a master regulator of cellular stress responses, relatively little is known about its regulation by SGs. When a transcriptome analysis of SGs utilizing U-2OS cells stably expressing G3BP1–GFP was carried out, p53 mRNA was not found to be enriched (Khong et al., 2017). In an analysis of the mRNAs that interact with the RBP TIA-1, the dissociation of p53 mRNA in response to a DNA damage reagent was reported in activated B cells (Diaz-Munoz et al., 2017). Notably, increased USP10 in prostate cancer cells inhibits the p53 signaling pathway via interaction with G3BP2 (Takayama et al., 2018).

Although our work mainly focused on cytosolic hCdc73, many studies on hCdc73 address its role as a component of the PAFc in the nucleus. PAFc associates with RNAPII and functions as a protein scaffold via interactions with various transcription factors and transcriptional regulators (Jaehning, 2010; Van Oss et al., 2017). In the nucleus, spatiotemporal regulation of enhanceosomes that are undergoing LLPS functions in the dynamic recruitment of a transcriptional regulatory network (Boija et al., 2018; Shrinivas et al., 2019). Co-activators and other regulatory factors of transcription form biomolecular condensates, the compartmentalization of which is driven by dynamic low-specificity interactions between IDRs and DNAs within the complex (Boija et al., 2018; Lu et al., 2020). In addition, the progression of RNAPII from the initiation stage to the elongation stage depends on its phase separation properties, which are controlled by hyperphosphorylation of the intrinsically disordered C-terminal domain (Lu et al., 2018). Discovery of the phase separation properties of hCdc73 mediated by its IDR therefore opens the possibility of the involvement of hCdc73 or PAFc within nuclear biomolecular condensates with transcriptional activities. We also postulate that hCdc73 might be found in various molecular condensates in the nucleus, in addition to cytosolic SGs. Notably, nuclear hCdc73 has been reported to be present within amyloid bodies, physiological amyloid aggregates that form within the nuclei of stressed cells (Marijan et al., 2019).

In summary, here, we report the translocation of hCdc73 to SGs under various cellular stress conditions. We believe that the sequestration of hCdc73 might represent a novel way to control the mRNA stability of the stress-responsive p53 gene. Although we do not know how p53 mRNA is selectively targeted by cytosolic hCdc73, we are interested in determining how this selectivity is achieved and the repertoire of mRNAs that are controlled by this mechanism. As the activities of both nuclear and cytosolic hCdc73 are closely linked to carcinogenesis, it is also necessary to understand the physiological consequences of hCdc73 translocation to SGs and its relationship with tumorigenesis.

## MATERIALS AND METHODS

### Cell culture, transfection and viability assay

HeLa, HEK-293, HEK-293 T and U-2OS (ATCC, Manassas, Virginia, USA) cells were grown in Dulbecco's modified Eagle's medium (DMEM; Lonza, Basel, Switzerland) supplemented with 10% fetal bovine serum (FBS; Welgene, Taipei, Taiwan) and routinely checked for mycoplasma contamination with a mycoplasma PCR detection kit (iNTRON, Seongnam, Korea). Lipofectamine 2000 (Invitrogen, Waltham, Massachusetts, USA) or polyethyleneimine (PEI; Polysciences, Warrington, Pennsylvania) was used for transient transfection of short interfering RNA (siRNA) or plasmid DNA, respectively, according to the manufacturer's recommendations. For plasmid DNA, cells were harvested or fixed for further analysis after 48 h of transfection. For siRNA transfection, we used pooled siRNAs to increase the knockdown efficiency. For hCdc73, G3BP1, G3BP2, FXR1 and UBAP2L, two different kinds of siRNAs were used. For FMR1 and NUFIP2, three different kinds of siRNAs were used. For CAPRIN1 and USP10, four different siRNAs were used. For detailed information on the siRNAs, see Table S1. When pooled siRNAs (two, three, or four siRNAs) were used, we set the total amount to 80 pmole for each transfection seeded in six-well plates. At 72 h post-transfection, cells were treated with sodium arsenite (as described in figure legends) or fixed for further analysis. For reconstitution experiments, we performed double-round transfection. First, siRNAs for target protein were transfected with Lipofectamine 2000 and incubated for 24 h. Then, plasmid DNAs were transfected with PEI, followed by 48 h of incubation before treatment with drugs, harvest, or fixation. For the cell viability check, cells were mixed with 0.4% Trypan Blue at a ratio of 1:1 and incubated for 3 min at room temperature. Cell viability was measured within 3 to 5 min using CellDrop BF (DeNoVIX, Wilmington, Delaware).

### Plasmids, siRNAs and oligomers

hCdc73–Myc and GST–hCdc73 have been described previously (Jo et al., 2014). To generate mutant forms of hCdc73, site-directed mutagenesis and Gibson assembly (NEB, Ipswich, Massachusetts, USA) were performed. In particular, IDR deletion mutants were cloned from hCdc73^NLSmut^-containing plasmids. For the hCdc73 reconstitution experiment, we introduced silent mutations within the siRNA targeting site of hCdc73 plasmids to avoid degradation (5′-TACATGGTAAAGCAT-3′ to 5′-TATATGGTGAAGCAC-3′). optoDroplet plasmids (a gift from Yongdae Shin, Department of Chemistry, Seoul National University, Seoul, Korea) were subcloned into pCDNA.3.1 (Thermo Fisher Scientific) for transient overexpression. pEBG-GST-hCdc73 mutants were subcloned from hCdc73^NLSmut^-Myc and hCdc73^NLSmut^-IDR mutants-Myc. To generate FLAG-tagged FMR1, CAPRIN1 and USP10, HEK-293 cDNA was PCR amplified and cloned into pFLAG-CMV (Sigma-Aldrich). siRNAs were synthesized by Genepharma (Shanghai, China). The siRNA and qRT-PCR primer sequences used in this study are provided in Table S1.

### Immunoblotting

For western blot analysis, cells were lysed in lysis buffer (150 mM NaCl, 1% Triton X-100, 0.1% SDS, and 0.5% deoxycholic acid with protease inhibitors, 25 mM Tris-HCl pH 7.5), and proteins in the total cell lysate were quantified using the Bradford method and separated on SDS-polyacrylamide gels at various percentages (7–12%). The proteins were transferred to nitrocellulose membranes and analyzed with the indicated antibodies (see below for source and dilutions). Finally, the membranes were incubated with horseradish peroxidase (HRP)-conjugated secondary antibodies, and an LAS4000 luminescent image analyzer (Fujifilm, Tokyo, Japan) was used to visualize immunoreactive signals. The signals were acquired using SuperSignal West Femto Maximum Sensitivity substrate (Thermo Fisher Scientific, Waltham, Massachusetts, USA). We provide the raw western blot data in Fig. S6.

### Cellular fractionation

For cytoplasmic and nuclear fractionation, 5×10^6^ cells were resuspended in sucrose buffer (320 mM sucrose, 3 mM CaCl_2_, 2 mM MgOAc, 0.1 mM EDTA, 0.5% Nonidet P-40, 1 mM dithiothreitol, 0.5 mM phenylmethyl sulfonyl fluoride, and 10 mM Tris-HCl, pH 8.0). After centrifugation at 600 ***g***, the cytoplasmic (supernatant) fraction was removed. The pellet was washed with sucrose buffer without Nonidet P-40 and lysed in lysis buffer. The mixture was incubated on ice for 30 min and centrifuged at 13,000 ***g***. The supernatant, which contained the soluble nuclear proteins, was removed. The pellet was boiled with Laemmli sample buffer (Bio-Rad, Hercules, California, USA) supplemented with 5% β-mercaptoethanol at 95°C for 10 min to obtain the insoluble fraction.

### Immunofluorescence microscopy and quantification

Cells were cultured on round cover glasses in six-well culture dishes and washed with PBS, followed by fixation with cold methanol or 4% paraformaldehyde. The cells were then permeabilized by incubation with 0.2% Triton X-100 for 15 min and blocked with 5% BSA in PBST. The cells were incubated with primary antibodies overnight at 4°C, and then secondary antibodies conjugated to Alexa Fluor 488, 568, or 647 (Invitrogen) in 1% BSA-containing PBST were incubated with the cells for 1 h at room temperature. Then, a DAPI solution (Invitrogen) was used to visualize nuclei. Cover glasses were mounted on glass slides using fluorescence mounting medium (Agilent, Santa Clara, California) and analyzed with a FLUOVIEW FV3000 confocal microscope (Olympus, Tokyo, Japan) and TCS SP5 confocal microscope (Leica, Wetzlar, Germany). The area and number of SGs were determined using the ImageJ ‘Analyze particles’ plugin. The SG enrichment score was measured to quantitate the colocalization of hCdc73 and SGs. The area of SGs in the confocal microscopy images was set as the region of interest (ROI), and the fluorescence intensity of hCdc73 mutants within the ROI[SGs] was measured. Background ROI[BG] that exclude ROI[SGs] and represent the overall expression patterns of hCdc7 mutants in the cell were further designated. Next, the ‘SG enrichment score’ was calculated by dividing the intensity of hCdc73 mutants within ROI[SGs] by the intensity within the ROI[BG]. For imaging siRNA-transfected cells, BLOCK-iT Fluorescent Oligo for Lipid Transfection (Invitrogen) was co-transfected with the indicated siRNAs. Only fluorescent oligo-expressing cells were regarded as siRNA-transfected cells.

### Purification of the hCdc73-IDR proteins

All purified proteins were expressed from the pET28a vector in BL21(DE3) *Escherichia coli* cells. Expression cultures were grown in LB broth with constant shaking (200–240 rpm) at 37°C for 10 h. The bacteria were incubated in culture medium at 18°C for 36 h to allow bacterial protein expression. Bacterial pellets were resuspended in binding buffer (20 mM Tris-HCl pH 7.9, 500 mM NaCl, 10% glycerol, 7 mM β-mercaptoethanol, 8 M urea, and 25 mM imidazole) containing an EDTA-free protease inhibitor cocktail (Roche, Basel, Switzerland), followed by sonication to induce lysis. The supernatant obtained after centrifugation at 27,000 ***g*** for 30 min was purified using a HisTrap^TM^ HP column (GE Healthcare, Chicago, Illinois, USA). Next, the eluted proteins were dialyzed in dialysis tubing (Thermo Fisher Scientific) against a storage buffer (50 mM Tris-HCl, 0.5 M NaCl, 10% glycerol and 7 mM β-mercaptoethanol, pH adjusted to 7.4). The proteins were further purified using size exclusion chromatography (Superdex 200 Increase 10/300 GL; GE Healthcare) to remove nonspecific proteins. Vivaspin^®^ concentrators (Sartorius, Göttingen, Germany) were used to increase the concentration of the protein solution, which was subsequently stored at −80°C.

### *In vitro* phase separation assay

To assess LLPS, purified hCdc73-IDR proteins (10 μM) were mixed with LLPS buffer (150 mM NaCl, 10% PEG8000 and 50 mM Tris-HCl pH 7.4). The salt concentration and pH were changed as indicated, and the effects of these parameters on hCdc73-IDR(N+C) droplet behavior were observed. For this, 2 μl of protein mixed with LLPS buffer was loaded on 3% BSA-coated glass slides (Paul Marienfeld, Lauda-Königshofen, Germany) and covered with a 10ø cover slip (Deckglaser) to observe LLPS droplets. Images of the droplets were taken immediately after the proteins were mixed with LLPS buffer using an Axiovert 200 M microscope (Zeiss, Oberkochen, Germany) or a FLUOVIEW FV3000 confocal microscope (Olympus). ZEN 3.0 or FV31S-DT software was used to analyze the images. To separate the supernatant and pellet according to the pH, purified protein was dialyzed in the pH buffer described below. Samples were centrifuged at 16,000 ***g*** for 1 min. The supernatant and pellet were separated and denatured in Laemmli buffer at 95°C for 10 min for SDS–PAGE, followed by Coomassie Blue staining or immunoblotting. Buffer at pH 5 (20 mM sodium acetate, 150 mM, pH 5), at pH 7.4 (20 mM sodium phosphate, 150 mM NaCl, pH 7.4), or at pH 9 (20 mM Tris-HCl, 150 mM NaCl, pH 9) was used.

### optoDroplet assay

All images of optoDroplet plasmid-transfected HeLa cells were captured using a 40× objective and TCS SP5 confocal microscope (Leica) to observe IDR behavior upon blue light activation (Shin et al., 2017). The intracellular distributions of the indicated IDRs were examined using a 561-nm laser following activation with blue light (488-nm laser). As a control, empty-mCherrry-CRY2-expressing cells were imaged with a 561-nm laser upon blue light activation (488-nm laser) for a maximum of 210 s. Afterward, optoDroplet-formed cells were quantified.

### Immunoprecipitation and GST pulldown assay

For immunoprecipitation, HEK-293 T, HeLa and U-2OS cells were lysed with NP-40 lysis buffer (20 mM Tris-HCl at pH 7.4, 150 mM NaCl, 2 mM EDTA, and 1% Nonidet P-40) or polysome lysis buffer (PLB; 10 mM HEPES at pH 7, 100 mM KCl, 5 mM MgCl_2_, and 0.5% Nonidet P-40) (Jo et al., 2014). A total of 1 mg of cell lysate was incubated with mouse normal IgG or G3BP1 antibody (BD, Franklin Lakes, New Jersey, USA; 611126) for 8 h with protein A/G agarose beads (Sigma-Aldrich, Burlington, Massachusetts, USA) at 4°C. For the GST pulldown experiments, 1 mg of cell lysate was incubated with 40 μl of glutathione–Sepharose beads (GE Healthcare) at 4°C. The beads were then washed with lysis buffer five times, and proteins were eluted from the beads by incubation with Laemmli sample buffer supplemented with 5% β-mercaptoethanol at 95°C for 10 min. All the inputs were quantified using the Bradford method and loaded in equal amounts.

### RNA isolation and real-time RT-PCR

Total RNA was extracted using RNAiso Plus (Takara Bio, Shiga, Japan). RNA (1 μg) was reverse transcribed with ImProm-II Reverse Transcriptase (Promega, Madison, Wisconsin) using oligo(dT) or random primers (Promega). PCR analysis of the cDNA was performed on a StepOne Plus Real-Time PCR System (Applied Biosystems, Waltham, Massachusetts, USA). In most of the PCR experiments, hRPL32 was used as the internal control. To inhibit transcription, 100 μM DRB (Sigma-Aldrich) was added for 12 h.

### FISH probes

The following FISH probes were purchased from LGC Biosearch Technologies (Lystrup, Denmark): TP53 (Stellaris^®^ FISH Probes, Human TP53 with Quasar^®^ 670 Dye) and CDK6 (Stellaris^®^ FISH Probes, Human CDK6 with Quasar^®^ 570 Dye). FISH was performed according to the manufacturer's recommendations (Stellaris^®^ RNA FISH Protocol for Adherent Cells; https://biosearch-cdn.azureedge.net/assetsv6/bti_stellaris_protocol_adherent_cell.pdf).

### RNA-IP assay

Cells were either crosslinked with 0.03% formaldehyde (HeLa) or not crosslinked (HEK-293); afterward, RNA-IP was performed as previously described previously (Jo et al., 2014). Approximately 5×10^7^ cells were lysed in PLB (100 mM KCl, 5 mM MgCl_2_, 10 mM HEPES, pH 7.0, and 0.5% Nonidet P-40) supplemented with protease inhibitors and 100 Uml-1 RNase inhibitor. After centrifugation at 3000 ***g***, the supernatant was precleared and then incubated with anti-hCdc73 antibody (Bethyl, Montgomery, Texas, a300-170a) and protein A/G agarose beads for 4 h at 4°C. After five washes with 1 ml of PLB, the bound complexes were eluted, and RNA was isolated from the eluate using RNAiso Plus (Takara).

### Antibodies and reagents

Reagents: sodium arsenite, thapsigargin, MG132 and 1,6-hexanediol were purchased from Sigma-Aldrich.

Antibodies against hCdc73 (Bethyl, a300-170a) was used for immunoblotting (1:20,000) and immunoprecipitation (1:1000). For immunofluorescence microscopy of hCdc73, antibodies from Invitrogen (PA5-26189; 1:100) and Santa Cruz Biotechnology (Dallas, Texas, sc-33638; 1:100) were used. SG marker antibodies against G3BP1 (BD, 611126; 1:200), TIA-1 (Abcam, Cambridge, UK, ab40693; 1:200), and TIAR (Cell Signaling Technology, Danvers, Massachusetts, USA, 5137; 1:100) were purchased as indicated. Primary antibodies against the following were used: GAPDH (Santa Cruz Biotechnology, sc-47724; 1:10,000), LAMINB (Santa Cruz Biotechnology, sc-374015; 1:1000), Myc (Santa Cruz Biotechnology, sc-40; 1:1000), GST (Santa Cruz Biotechnology, sc-138; 1:5000), Vinculin (Santa Cruz Biotechnology, sc-55465; 1:1000), eEF1Bγ (Santa Cruz Biotechnology, sc-393378 and Abcam, ab72368; 1:1000) and Flag (Sigma-Aldrich, F7425; 1:1000).

### Statistical analysis

All statistical analyses were performed and graphed using GraphPad Prism (version 9.2.0). The significance of differences was evaluated by an unpaired two-tailed *t*-test or one-way ANOVA with multiple comparisons. All data are presented as the mean±s.d. At least three biologically independent experiments were conducted to perform statistical analysis unless otherwise noted in the corresponding figure legend. In the case of quantification of imaging data, at least 10 cells per experiment were randomly taken for quantification. At least three biologically independent experiments were performed. Significance is defined as *P<0.05; **P<0.005; ***P<0.0005; ****P<0.00005; n.s., not significant.

## Supplementary Material

10.1242/joces.260593_sup1 Supplementary information
