# Supplementary material for: Translocation of cytosolic human Cdc73 to stress granules plays a role in arsenic stress-induced stabilization of p53 mRNA
Source: J Cell Sci. 2023 Jul 20;136(14):jcs260593. doi: 10.1242/jcs.260593 (PMC10399996; doi:10.1242/jcs.260593)
Supplement: Supplementary information [file joces-136-260593-s1.pdf]

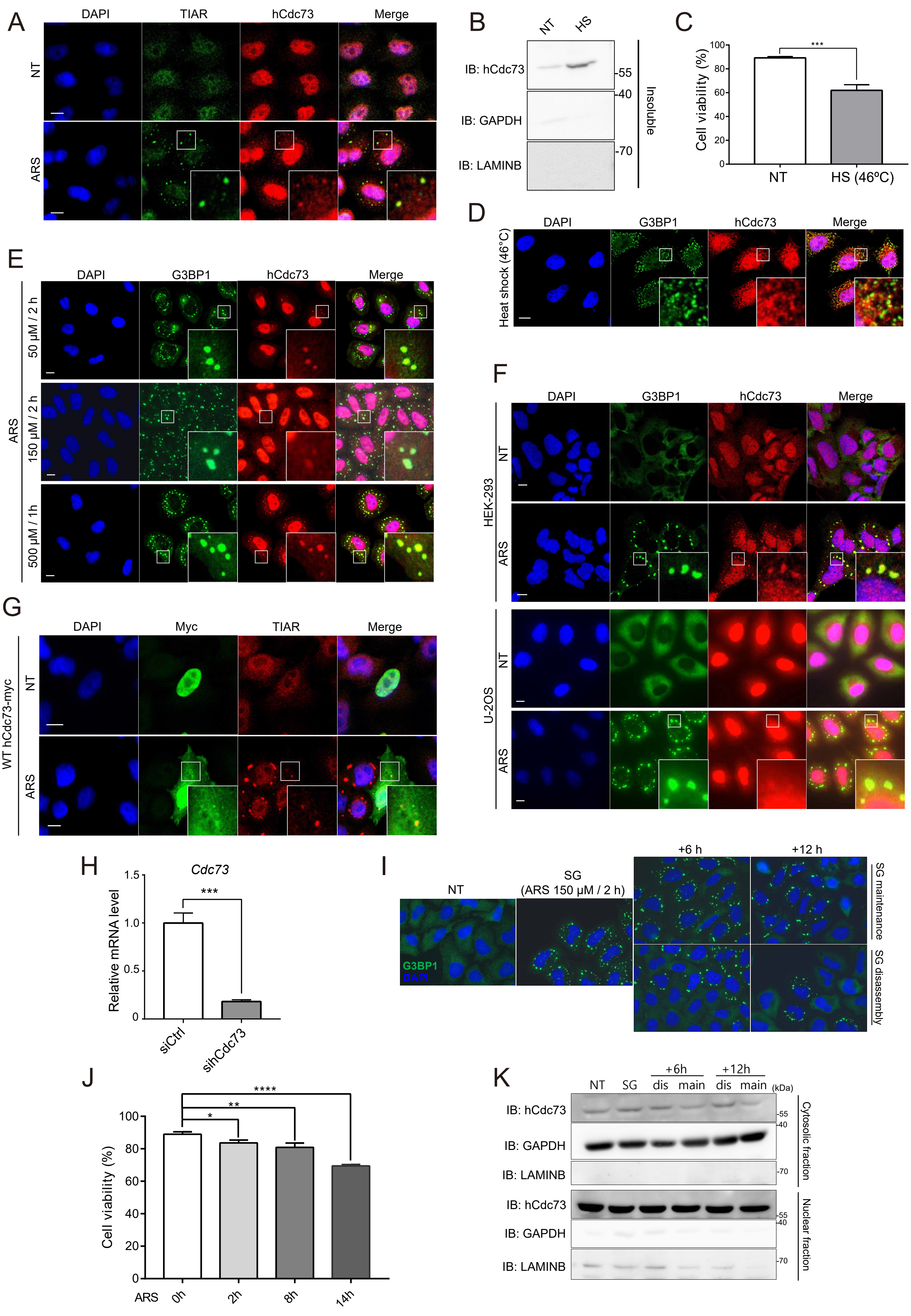


Journal of Cell Science • Supplementary information

**Fig. S1.** (A) Confocal images of endogenous hCdc73 and TIAR. HeLa cells were either untreated (NT) or treated with 500 μM sodium arsenite for 1 h (ARS). (B) Immunoblot analysis (IB) of hCdc73 from the insoluble fraction of cells exposed to heat shock (HS: 46°C for 1 h). (C) Cell viability of HeLa cells stressed with 46°C heat shock for 1 hour was measured by trypan blue staining. (D) Confocal image of endogenous hCdc73 and G3BP1 in HeLa cells stressed with heat shock. (E) HeLa cells were treated with various concentrations of sodium arsenite (ARS: 50, 150, and 500 μM) for the indicated duration. Afterward, confocal images of endogenous hCdc73 and G3BP1 were taken. (F) Confocal images of HEK-293 and U-2OS cells treated with 500 μ M sodium arsenite (ARS) for 1 h. (G) Confocal images of WT hCdc73-myc-transfected HeLa cells that were untreated (NT) or treated with 500 μM sodium arsenite for 1 h (ARS). TIAR was used as marker for SGs. (H) Knockdown efficiency of sihCdc73 was measured by RT-qPCR, hRPL32 was used as the internal control. (I) Representative immunofluorescence microscopy images of SG formation in HeLa cells. Endogenous G3BP1 and DAPI were stained after +6 h and +12 h of exposure to SG maintenance and disassembly conditions. The experimental scheme is shown in Figure 1G. (J) Cell viability of HeLa cells stressed with 150 μM of ARS for indicated time were measured by trypan blue staining. (K) Immunoblot analysis of hCdc73 from the cytosol (top), nucleus (bottom). HeLa cells were treated with 150 μM ARS for 2h (SG), an additional 6 or 12 h of exposure to SG maintenance (main) or disassembly (dis) conditions. All scale bars in this figure, 10 μm. Each graph represents mean data from three independent experiments and error bars are presented as the mean ± standard deviation.

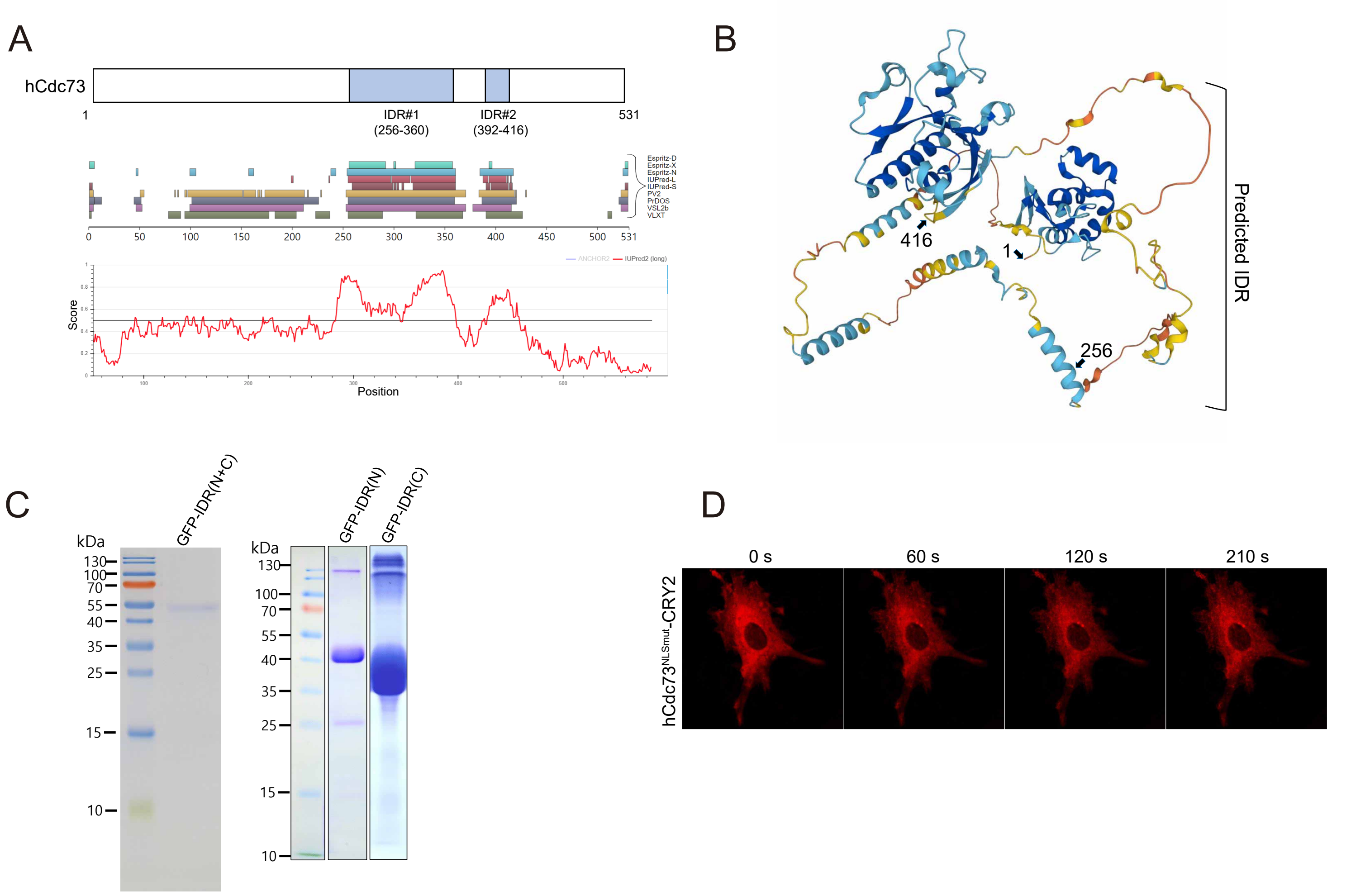


**Fig. S2.** (A) (top) Schematic diagram of the IDR region of hCdc73. 75 % agreement between all predictors in the D2P2 database for IDR region within hCdc73 being colored as blue. (middle) Various color boxed areas are predicted by multiple database resources that are listed in the right pannel accordingly. (bottom) Representative IDR score of hCdc73 obtained from the IUPred simulation program. The threshold scores for all predictors were 0.5. (B) Predicted 3D structure of hCdc73 from the AlphaFold (https://alphafold.ebi.ac.uk/entry/Q6P1J9). (C) Coomassie blue staining result of the purified 6X His-GFP-tagged-hCdc73-IDR(N+C), -IDR(N) and -IDR(C). (D) Time-lapse imaging of optoDroplet formation of hCdc73$^{NLSmut}$-mCherry-CRY2 upon blue light exposure in HeLa cells. Scale bar, 10 μm.

Journal of Cell Science • Supplementary information

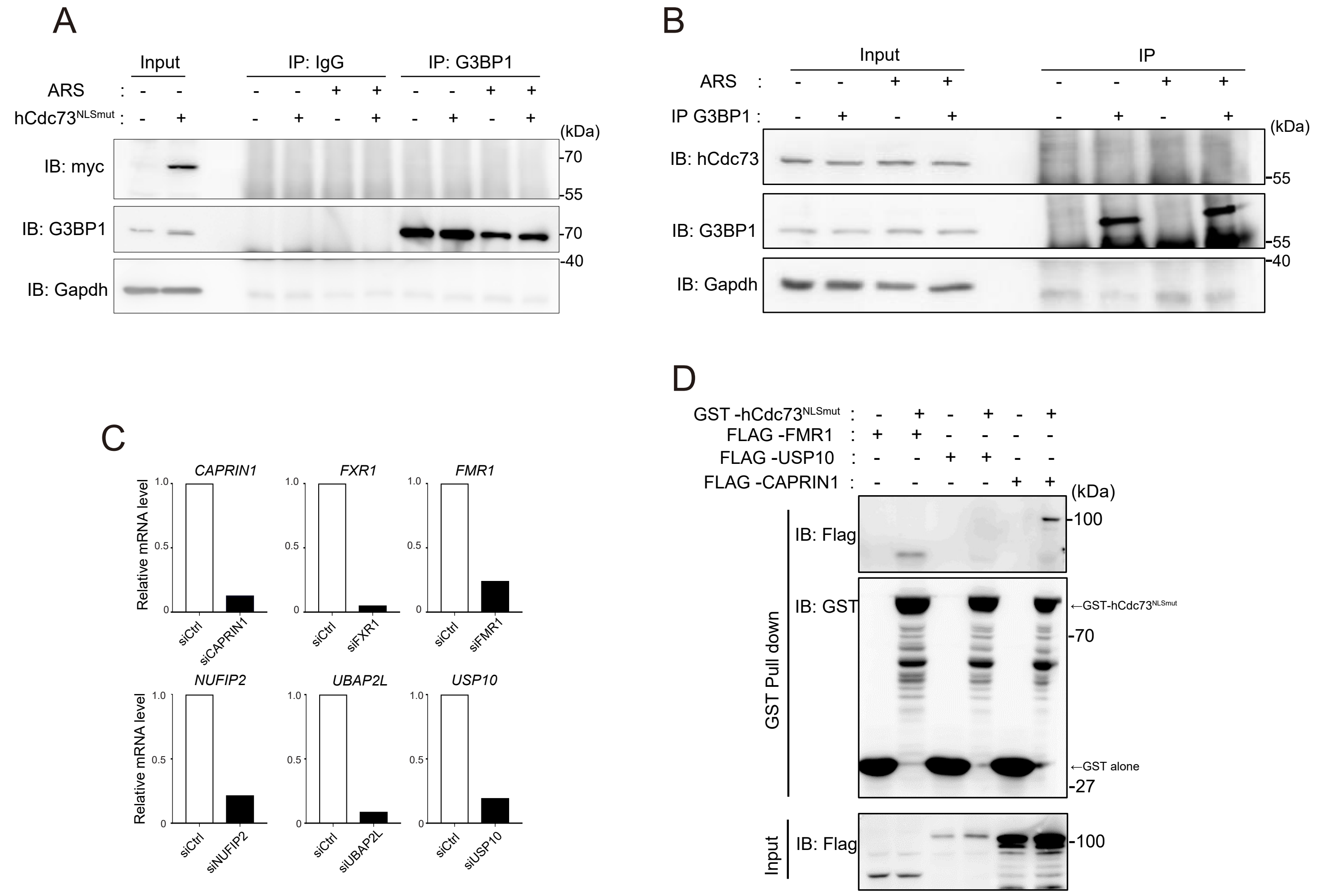


**Fig. S3.** To check the interaction between G3BP1 and hCdc73, IP assay was performed with hCdc73$^{NLSmut}$-myc and empty-myc (-). (A) and endogenous hCdc73 (B) with G3BP1 in HeLa cells. (C) The Knockdown efficiency of siRNA targeting stress granule scaffold proteins measured by RT-qPCR, hRPL32 was used as the internal control. (D) HEK-293T cells were transfected with the indicated plasmids and subjected to GST pulldown assay. ‘ - ’ indicates GST-empty or Flag-empty vector, respectively.

Journal of Cell Science • Supplementary information

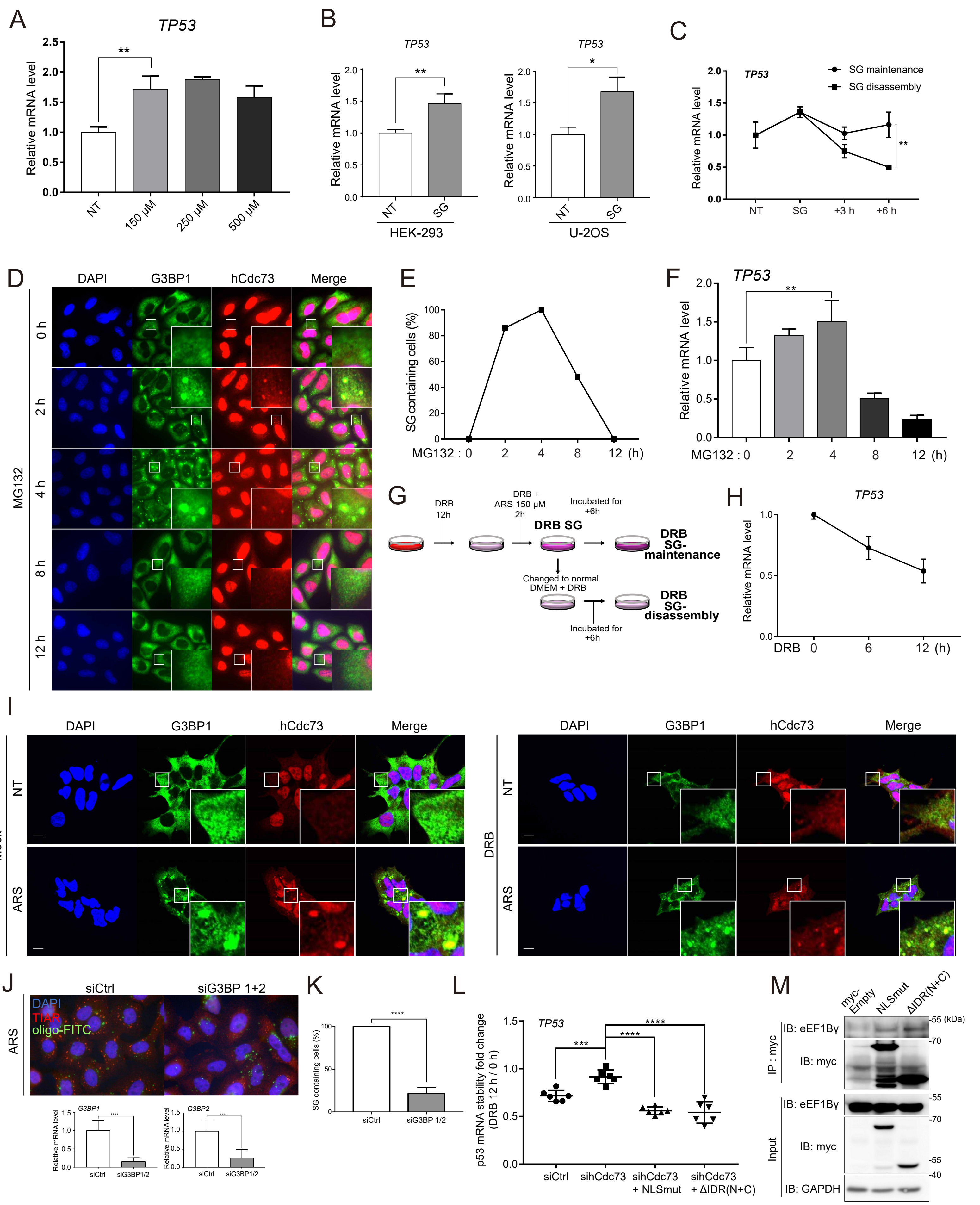


Journal of Cell Science • Supplementary information

**Fig. S4.** (A) HeLa cells were treated with 150, 250, or 500 μM sodium arsenite for 1 h. The p53 mRNA level in each stressed cell type was measured by RT-qPCR and compared with the NT (untreated) cells. (B) HEK-293 and U-2OS cells were stressed by treatment with 500 μM sodium arsenite for 1 h (SG). The p53 mRNA levels were measured by RT-qPCR. (C) HeLa cells were treated with 500 μM sodium arsenite for 1 h (SG) and then subjected to SG maintenance or disassembly according to the protocol shown in Figure 5B. At the indicated times, p53 mRNA levels were measured by RT-qPCR. (D) Representative immunofluorescence microscopy images of SG formation in HeLa cells. Endogenous G3BP1 and DAPI were stained after 2, 4, 8, 12 h of exposure to MG132 (10 μM). (E) SG containing cells are calculated from (D), and the p53 mRNA level was measured by RT-qPCR in (F). (G) Schematic diagram showing how experiments in Figure 5D,E have been performed. After 12 h of DRB pretreatment, the HEK-293 cells were stimulated with 150 μM sodium arsenite for 2 h, followed by SG maintenance or disassembly conditions along with DRB treatment. (H) HEK-293 cells were treated with 100 μM DRB for 6 and 12 h. The p53 mRNA level was measured after RT-qPCR. (I) Confocal images of endogenous hCdc73 and G3BP1. HEK-293 cells were treated with DMSO (Mock) or 100 μM DRB for 12 h (DRB). After DRB treatment, 150 μM sodium arsenite was added, as shown in Figure 5C. (J) (top) Representa-tive immunofluorescence microscopy images of siG3BP1 and siG3BP2 transfected HeLa cells. For the recognition of transfected cells, oligo-FITC was co-transfected. Endogenous TIAR (marker for SG) and DAPI were stained after the 150 μM sodium arse-nite treatment for 2 h. (bottom) The knockdown efficiency was measured by RT-qPCR. (K) Stress granule containing siCtrl and siG3BP1+2 transfected cells from (I) are quantitated. (L) hCdc73 siRNA-transfected HEK-293 cells are reconstituted with hCdc73$^{NLSmut}$-myc (NLSmut) or hCdc73NLS$^{mut}$-ΔIDR(N+C)-myc, ΔIDR(N+C). After transfection inhibition with DRB (100 μM, 12 h), the fold change in p53 mRNA stability (DRB 12 h compare to 0 h) were measured by RT-qPCR. hRPL32 was used as the internal control in all the RT-qPCR data above. (M) Immunoprecipitation assay was performed with hCdc73$^{NLSmut}$-myc and hCdc73$^{NLSmut}$- ΔIDR(N+C)-myc to check their interaction with endogenous eEF1Bγ in HEK- 293T cells. Each graph represents mean data from three independent experiments and error bars are presented as the mean ± standard deviation. Statistical values were calculated using an unpaired t test.

Journal of Cell Science • Supplementary information

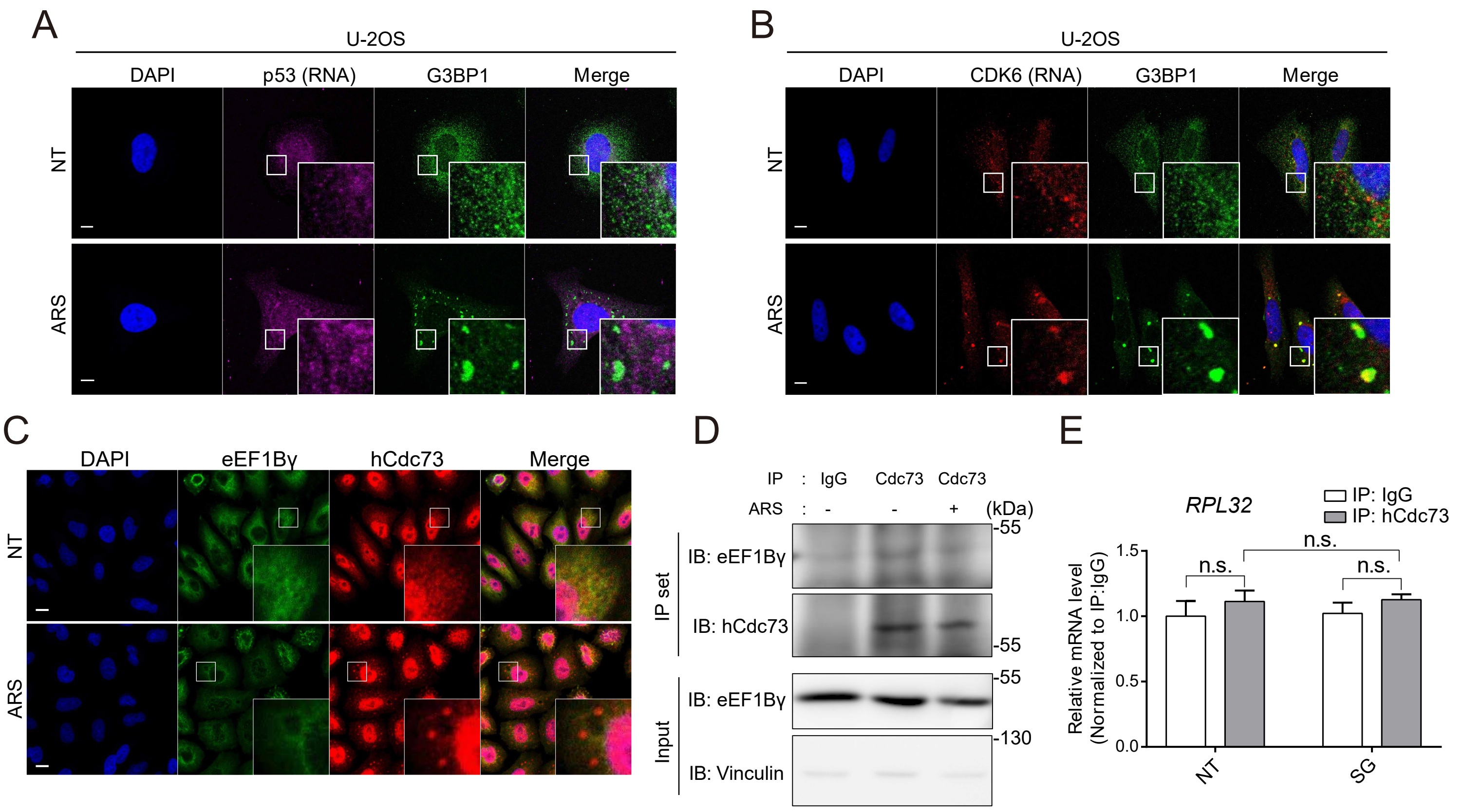


**Fig. S5.** (A, B) Confocal images from RNA FISH experiments. U-2OS cells were either untreated (NT) or treated with 500 μM sodium arsenite for 1 h. FISH probes that specifically bind p53 (A) and CDK6 (B) mRNA were used. (C) Representative confocal images of endogenous hCdc73 and eEF1Bγ. HeLa cells were untreated (NT) or treated with 500 μM of sodium arsenite for 1 h (ARS). All scale bars, 10 μm. (D) To check the interaction between eEF1Bγ and hCdc73 IP assay was performed with endog-enous hCdc73. U-2OS cells were either untreated or treated with 500 μM of sodium arsenite for 1h (ARS). (E) Negative control RNA-IP set of Figure 6D, RNA-IP performed using IgG or endogenous hCdc73 antibodies with the cytosolic fraction of HEK-293 cells. Left untreated (NT) or treated with 500 μM sodium arsenite for 1 h (ARS). Bound mRNA was measured by RT–qPCR with house keeping gene hRPL32.

Journal of Cell Science • Supplementary information

**Fig. S6.** Supplementary material: Blot transparency Figure 1A , (S1B)

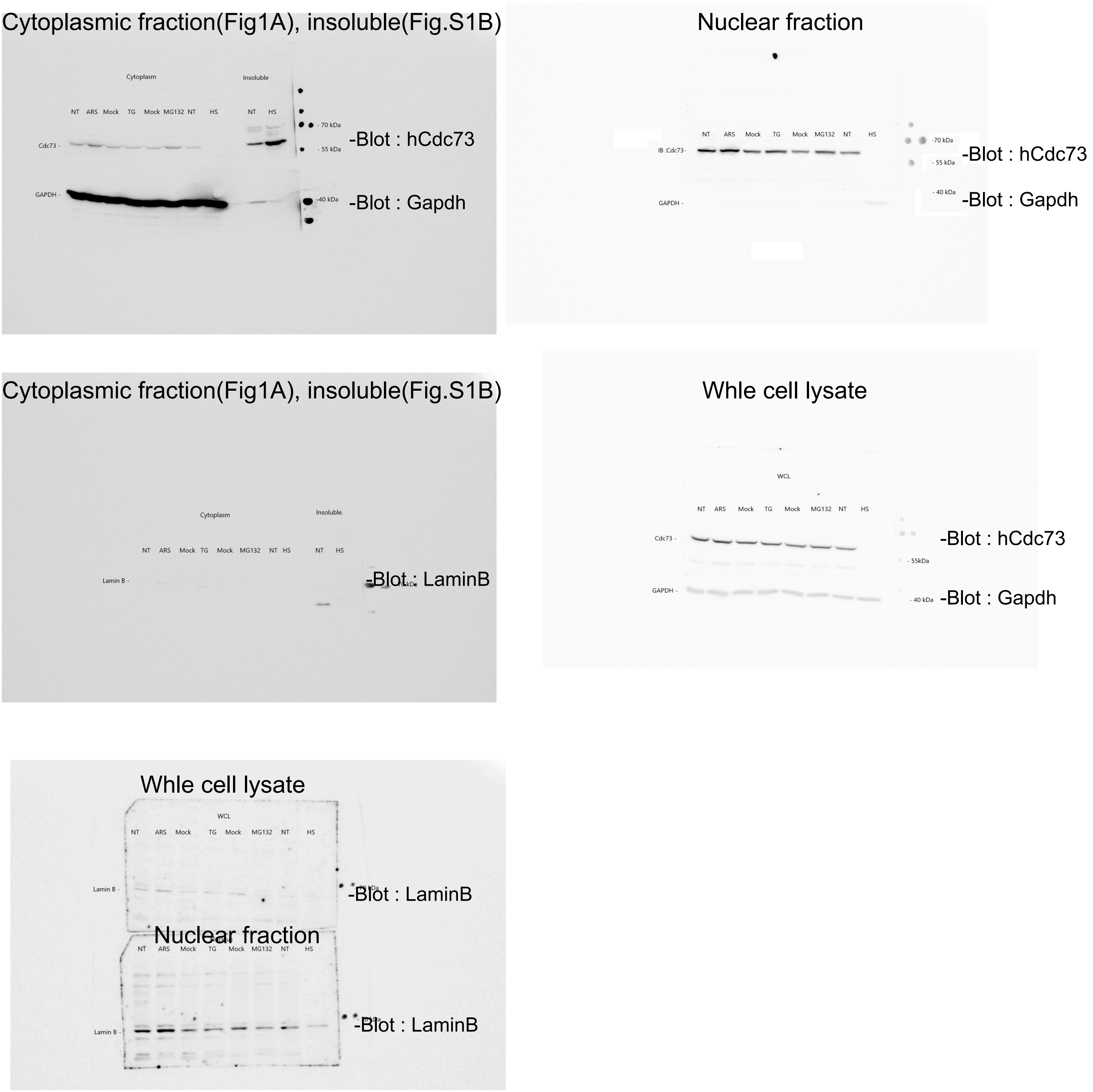


Journal of Cell Science • Supplementary information

**Fig. S6.** Blot transparency

# Figure 4E

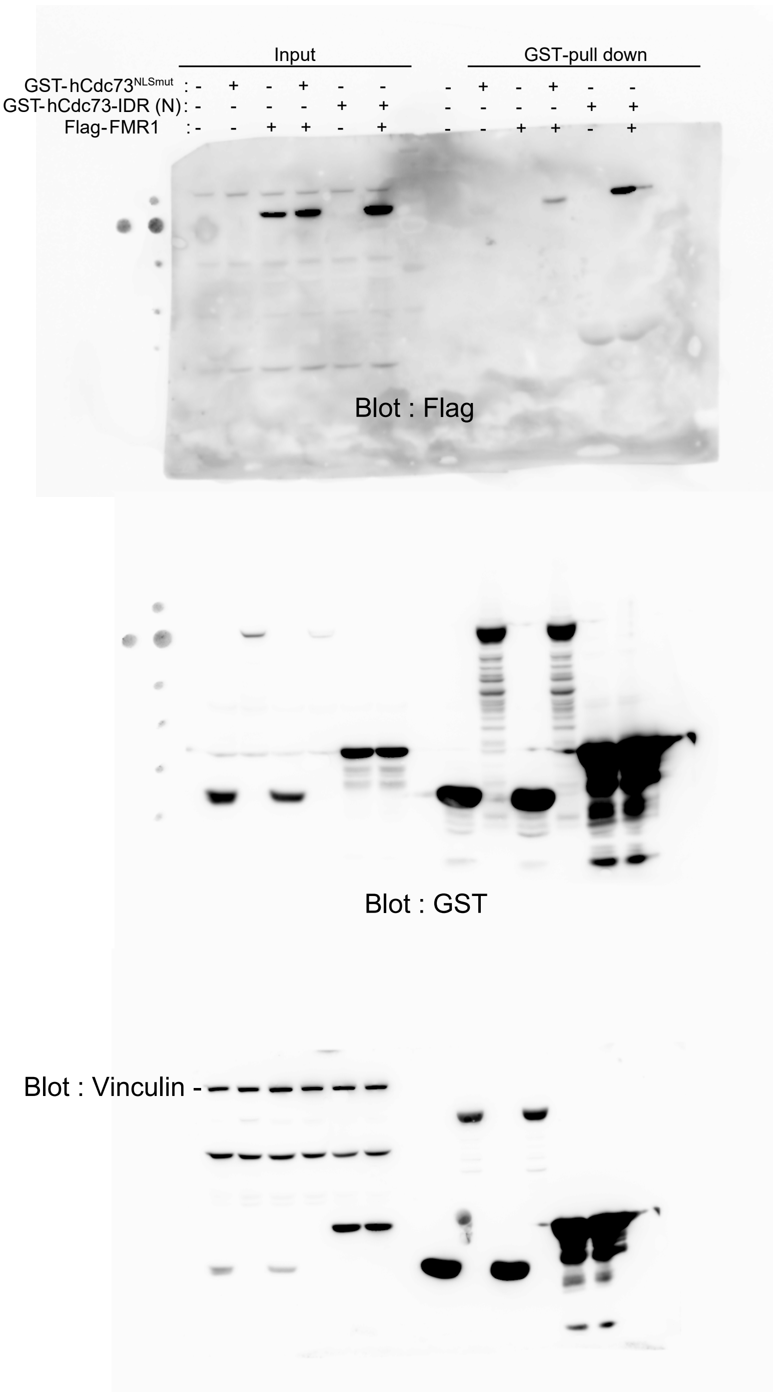


# Figure 4F

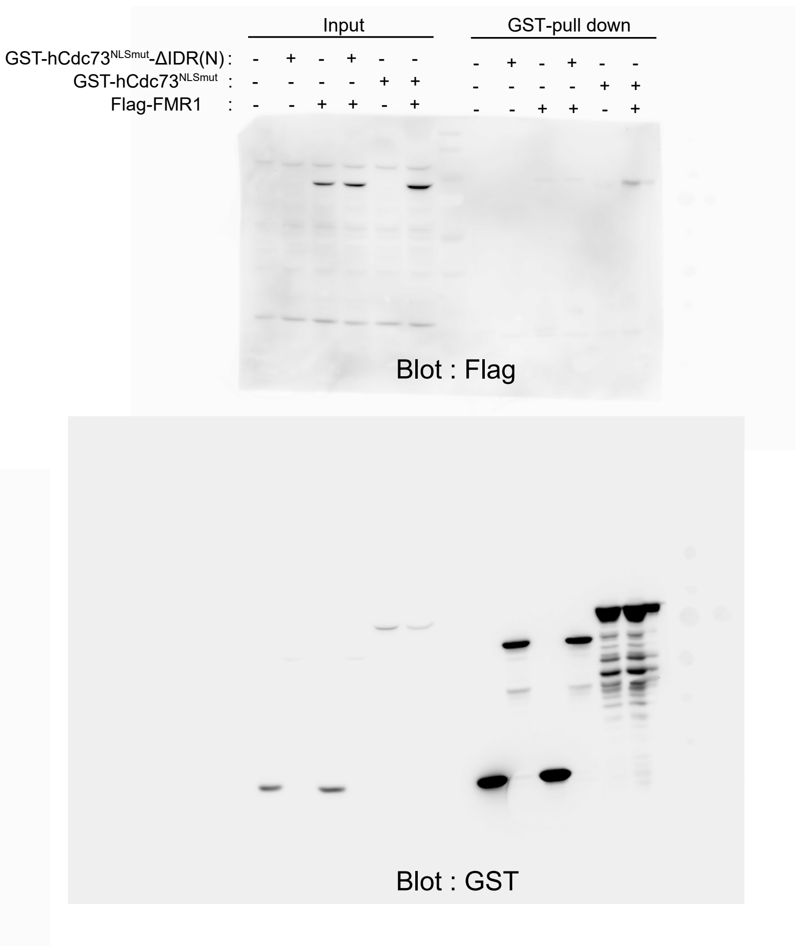


Journal of Cell Science • Supplementary information

**Fig. S6.** Blot transparency

# Figure 5C

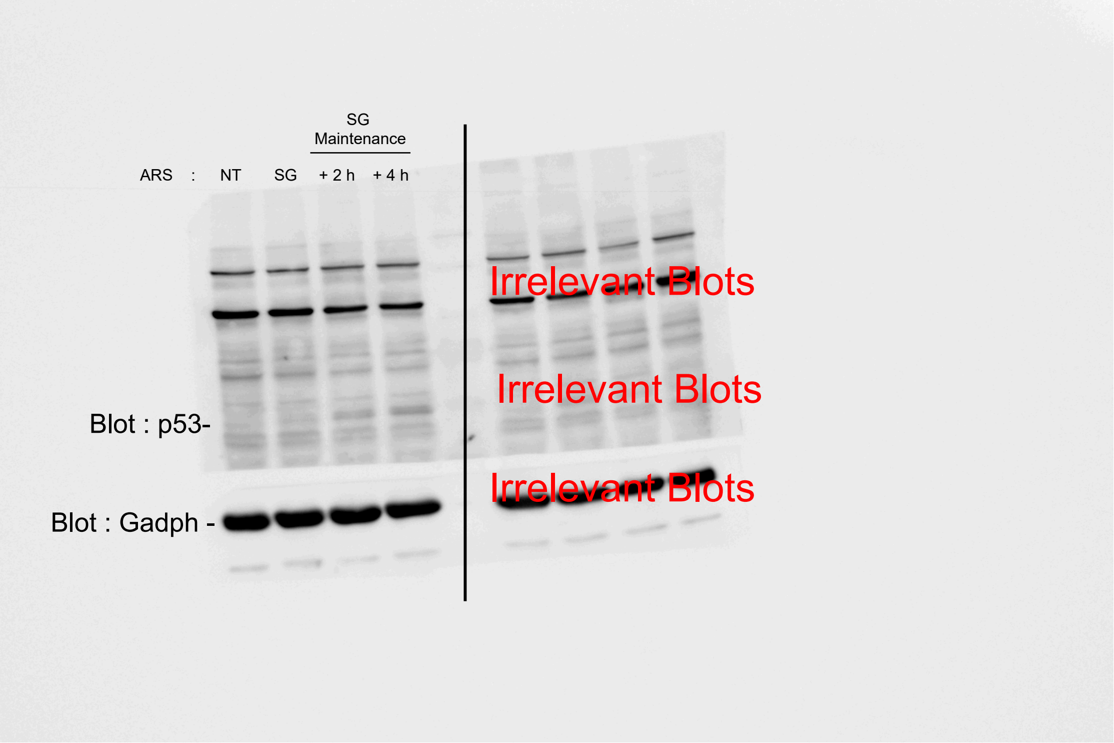

**Fig. S6.** Blot transparency

## Figure S1K

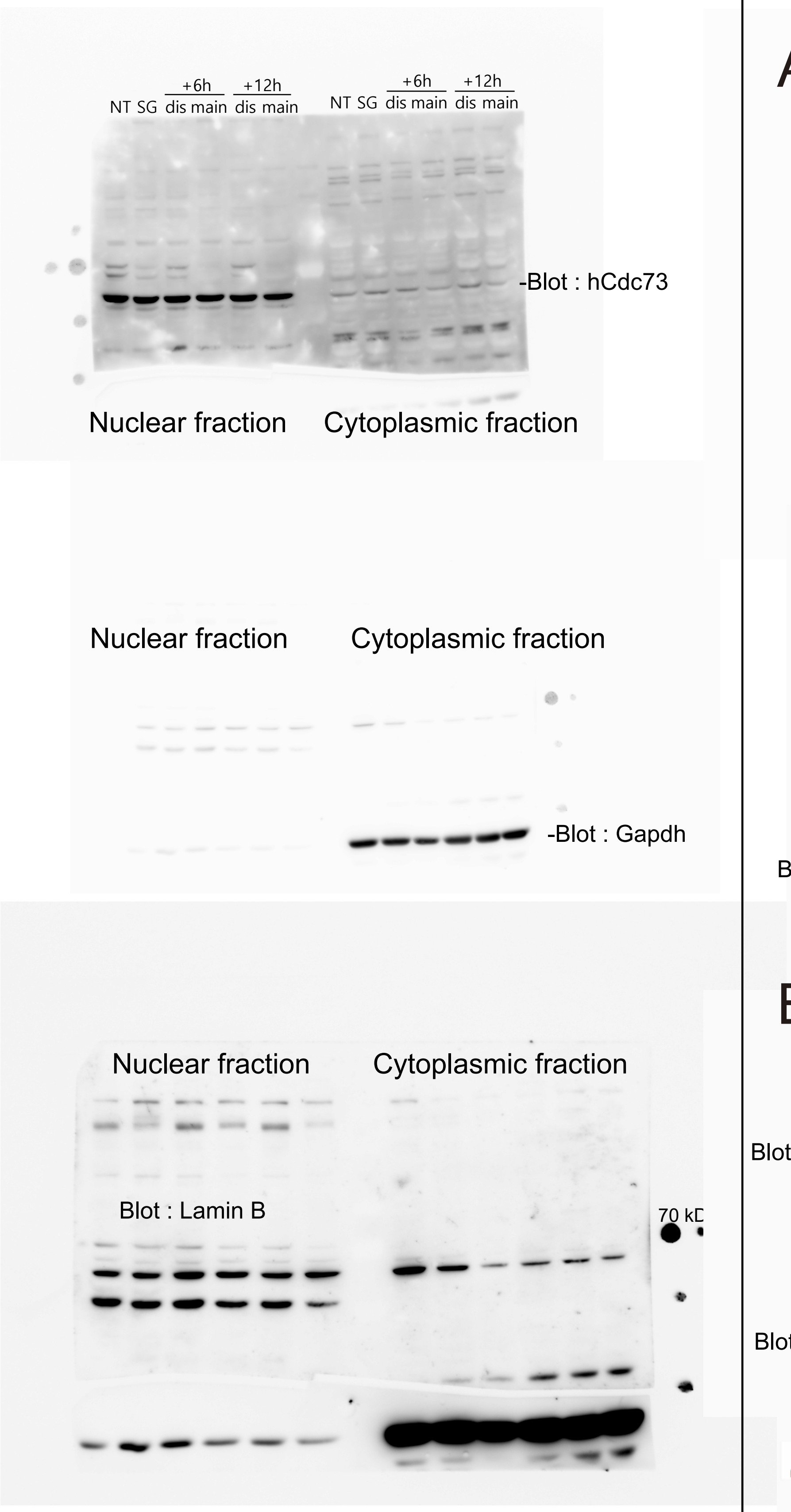


## Figure S3A,B

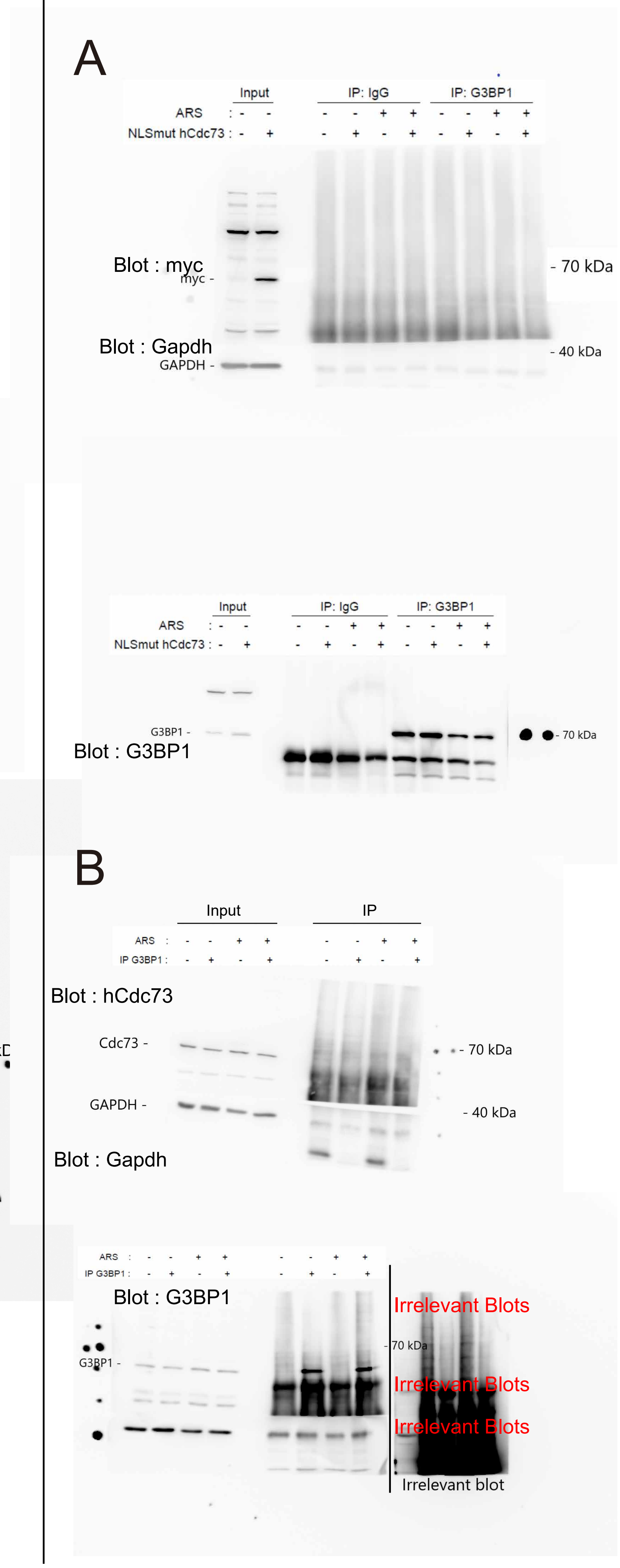


Journal of Cell Science • Supplementary information

**Fig. S6.** Blot transparency

# Figure S3D

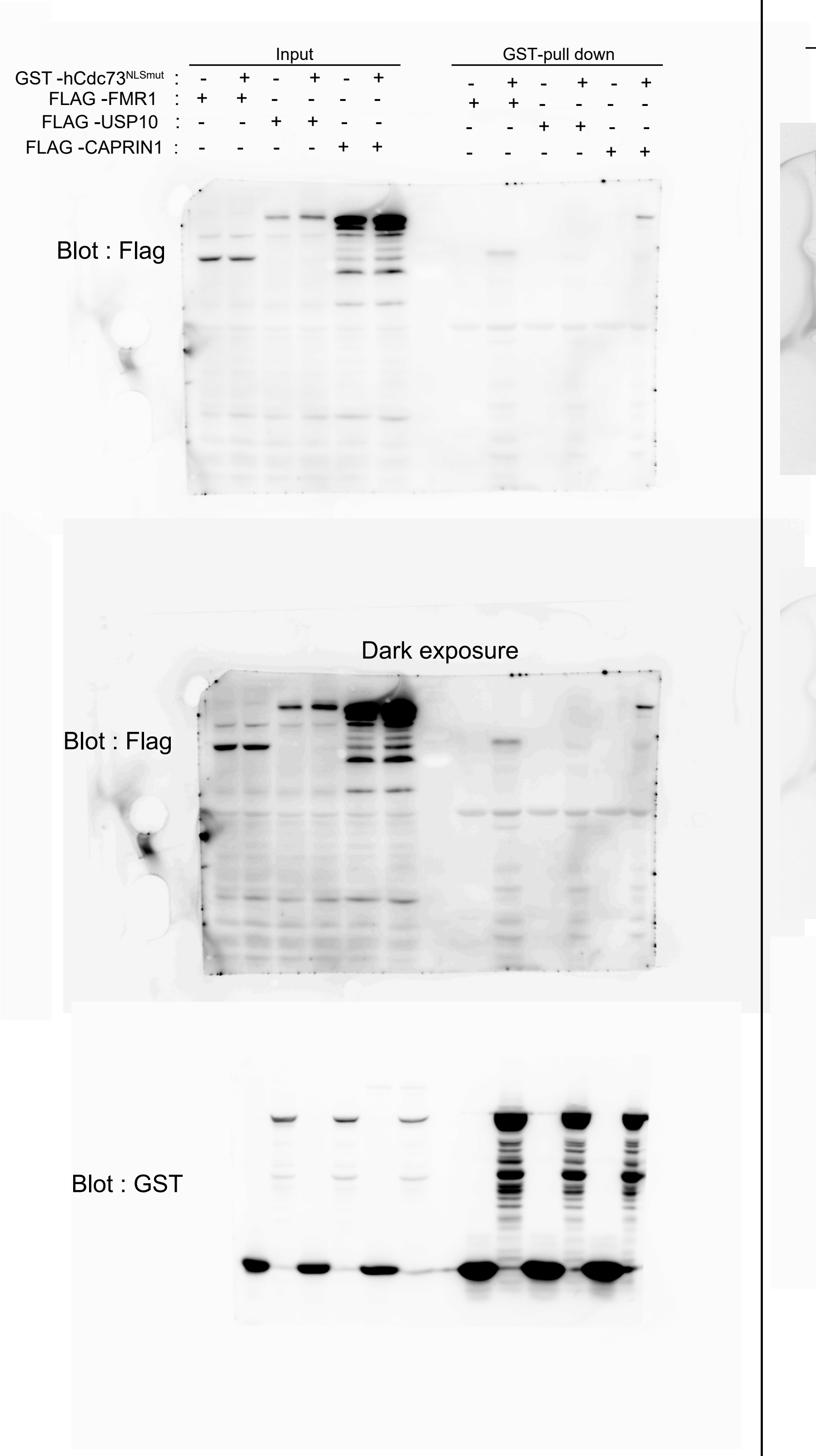


# Figure S4M

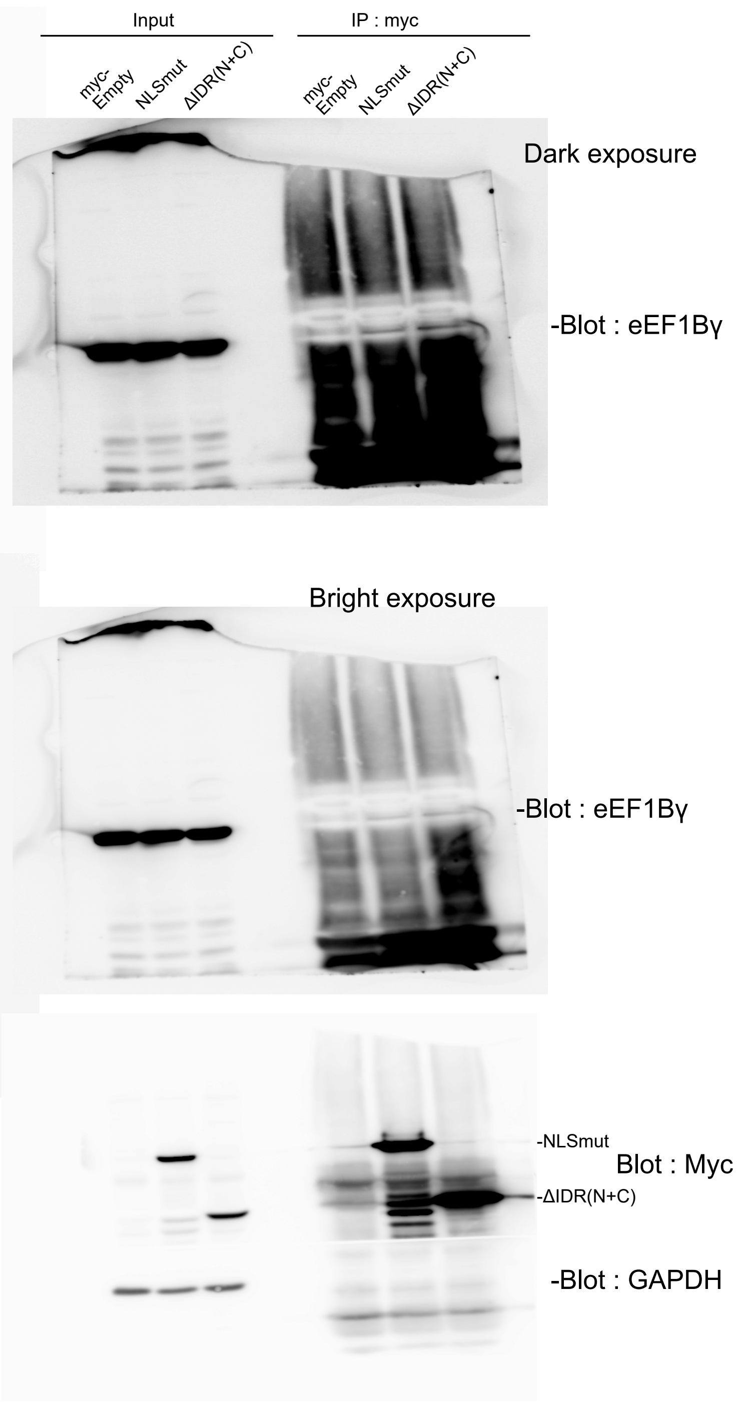


Journal of Cell Science • Supplementary information

# Figure S5E

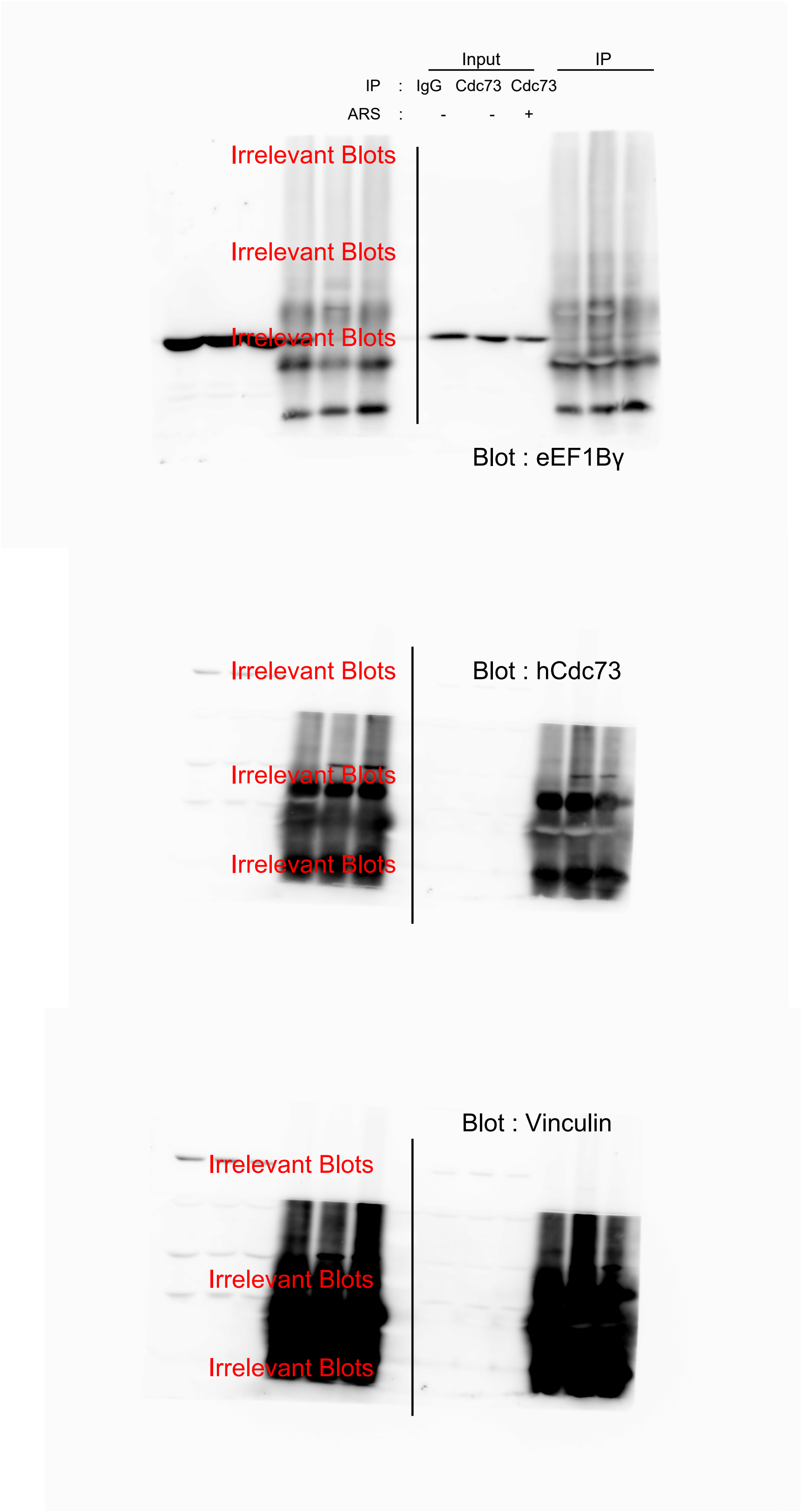


Journal of Cell Science • Supplementary information

**Table S1.** siRNA and primer sequence information used in this study

siRNA sequence ; synthesized from Genepharma (Shanghai, China)

| Name | Targeting sequence | Remark |
|---|---|---|
| sihCdc73 #1 | GGTACATGGTAAAGCATAARemark | CDS |
| sihCdc73 #2 | CAGCGATCTACTCAAGTCAAA | CDS |
| siUSP10 #1 | CCATAAAGATTGCAGAGTT | CDS |
| siUSP10 #2 | CAAACAAGAGGTTGAGATA | CDS |
| siUSP10 #3 | CCACATATATTTACAGACT | CDS |
| siUSP10 #4 | GAGTTGCACACCACGGAAA | CDS |
| siCAPRIN1 #1 | AGGGTAAGCTTGATGATTA | CDS |
| siCAPRIN1 #2 | GCACGTCGGGAGCAGCTTA | CDS |
| siCAPRIN1 #3 | GGAAATTGTTGAGCGTGTT | CDS |
| siCAPRIN1 #4 | TAGTCAGCCTCACCAAGTA | CDS |
| siUBAP2L #1 | GCCTGTCCTTTCTGATTAT | CDS |
| siUBAP2L #2 | GCCACAAGTATATGGTTAT | CDS |
| siFMR1 #1 | GGTGTATTCCAGAGCAAAT | CDS |
| siFMR1 #2 | GTACTGAGCAGTGATATTC | 3'-UTR |
| siFMR1 #3 | AAGTTGTATGATCTGTGCC | 3'-UTR |
| siFXR1 #1 | GCAATCCATACAGCTTACTTGATAA | CDS |
| siFXR1 #2 | GAAGTTGATGCTTATGTCCAGAAAT | CDS |
| siNUFIP2 #1 | AAGCAGACACCAGTAGTCAAG | CDS |
| siNUFIP2 #2 | AAGGCTTACGAGCTGGAGAAA | CDS |
| siNUFIP2 #3 | AAGCTCAGATAGTAAACCTGG | CDS |
| siG3BP1 #1 | GCCTGAGCCAGTATTAGAA | CDS |
| siG3BP1 #2 | CCTTCTGGTGTGGAGAAAT | CDS |
| siG3BP2 #1 | GACTCTGACAACCGTAGAATA | CDS |
| siG3BP2 #2 | GTGATGATCGCAGGGATATTA | CDS |

*Abbreviations : CDS ; Coding sequence , 3'-UTR ; 3'-Untranslated region

Journal of Cell Science • Supplementary information

Primers for RT-qPCR

| Name | Primer sequence (5’ to 3’) |
|---|---|
| hRPL32 | CAAGGGCCAGATCTTGATGCC |
| | GCGATCTCGGCACAGTAAGAT |
| hCdc73 | TTAGCGTCCTGCGACAGTACAACA |
| | TCACATTCTTGGGCCAGGAGAACT |
| CAPRIN1 | TCTCGGGGTGATCGACAAGAA |
| | CCCTTTGTTCATTCGTTCCTGG |
| FXR1 | AGCTGCGACAGATTGGTTCT |
| | TCAGAGGGGTTAGACAGCTCA |
| FMR1 | GCAGCATGTGATGCAACTTACA |
| | CGCCTCTTTGGCACACATT |
| NUFIP2 | GCAGAAGCAAGATCCCAAAAGG |
| | TTGTGTCCCACATGAACGGT |
| UBAP2L | ACACAATCCCCATCACTGGT |
| | CAGAGGAGAAGACGGAGGTG |
| USP10 | GGCTGTGGATAAACTACCTGAT |
| | GTGTGCTTGAAATACTGTAGCTG |
| TP53 | CCCTCTGAGTCAGGAAACATTTT |
| | CAGCATCAAATCATCCATTGCT |
| TP53-5’ (RNA-IP) | GCATTCTGGGACAGCCAAGTCTG |
| | TGAGGATGGGCCTCCGGTTCATGC |
| G3BP1 | CTTTGGGTTTGTCACTGAGC |
| | GGTGTTTGCTGTCTTTCTTCAGGTTCC |
| G3BP2 | GAGCTGAAACCACAAGTGGAGG |
| | GGTCACTGAAGCCCAGGAGAAA |

Journal of Cell Science • Supplementary information
