# Supplementary material for: Translocation of cytosolic human Cdc73 to stress granules plays a role in arsenic stress-induced stabilization of p53 mRNA
Source: J Cell Sci. 2023 Jul 20;136(14):jcs260593. doi: 10.1242/jcs.260593 (PMC10399996; doi:10.1242/jcs.260593)
Supplement: Supplementary file 1 [file joces-136-260593_review_history.pdf]

Translocation of cytosolic human Cdc73 to stress granules plays a role in arsenic stress-induced stabilization of p53 mRNA

Hojin Lee, Tae-Hyeon Kim and Joo-Yeon Yoo

DOI: 10.1242/jcs.260593

Editor: Maria Carmo-Fonseca

Review timeline

Original submission:	1 September 2022
Editorial decision:	3 October 2022
First revision received:	5 April 2023
Editorial decision:	9 May 2023
Second revision received:	14 June 2023
Accepted:	18 June 2023

Original submission

First decision letter

MS ID#: JOCES/2022/260593

MS TITLE: p53 mRNA availability during the cellular stress response is controlled by Cdc73 sequestration to stress granules

AUTHORS: Hojin Lee, Tae-Hyeon Kim, and Joo Yeon Yoo
ARTICLE TYPE: Research Article

We have now reached a decision on the above manuscript.

To see the reviewers' reports and a copy of this decision letter, please go to: https://submit-jcs.biologists.org and click on the 'Manuscripts with Decisions' queue in the Author Area. (Corresponding author only has access to reviews.)

I have now received comments on your manuscript from three expert referees. As you will see from their reports, all thought that the work is potentially quite interesting and significant but also raised a number of concerns that prevent me from accepting the paper. Although reviewer #2 considers that your study does not reach the standard to be published in JCS, I agree with reviewers #1 and 3 who recommend that a revised version of the manuscript can be reconsidered. If you think that you can deal satisfactorily with the criticisms on revision, I would be pleased to see a revised manuscript.

Please ensure that you clearly highlight all changes made in the revised manuscript. Please avoid using 'Tracked changes' in Word files as these are lost in PDF conversion.

I should be grateful if you would also provide a point-by-point response detailing how you have dealt with the points raised by the reviewers in the 'Response to Reviewers' box. Please attend to all of the reviewers' comments. If you do not agree with any of their criticisms or suggestions please explain clearly why this is so.

© 2023. Published by The Company of Biologists under the terms of the Creative Commons Attribution License (https://creativecommons.org/licenses/by/4.0/).

Reviewer 1

Advance summary and potential significance to field

In this manuscript, the authors demonstrate a posttranslational control of p53 mRNA levels mediated by selective sequestration of hCdc73 to stress granules under stress conditions. This work further details the authors' previous finding that showed hCdc73/eEF1Bγ/Ski8 complex-mediated p53 mRNA level control in the cytosol and provides a novel pathway of p53 mRNA level regulation by SGs. Overall, experiments are well designed, conducted, and supportive of conclusions. This reviewer raises the following issues.

Comments for the author

1. Throughout the manuscript, [Paf1 complex, PAF complex, PAFc], [RNAPII, RNA Pol II], and [hCdc73, Cdc73] are used as a mix. It would be good to unify terminology.
2. G3BP1 is stained green in most experiments. However, in some figures where G3BP1 is stained pale blue-green (e.g., Fig. 1E, 4A), it is hard to evaluate the colocalization of G3BP1 and Cdc73.
3. In Fig. S2, the figure legend for (top) does not match the top figure. Also a more detailed description for the middle panel is necessary.
4. What is TIA-1 used in Figure 2? The usage of this reagent is not described in Materials and Methods. Are TIAR and TIA-1 same reagent?
5. Inclusion of SDS-PAGE/Coomassie Blue staining of purified 6X His-GFP-tagged hCdc73-IDR(N+C) protein as a Supplemental Figure is recommended to demonstrate the quality of purified protein.
6. In Fig. 3A and 3B, indicate what DIC means in the figure legend. Is it Differential Interference Contrast?
7. In Fig 3, in addition to hCdc73 protein fragments, it is more interesting to test whether full-length hCdc73 has a phase separation property because endogenous hCdc73 is present as a full-length form in cells. In addition, an experiment with hCdc73 protein missing IDR(C) will provide a conclusive answer for the requirement of this region for phase separation property.
8. In Fig. 4, the inclusion of GST pull-down assays with (one or two) proteins that did not change the colocalization patterns (e.g., CAPRIN1, FXR1, NUFOP2 UBAP2L, or USP10) will strengthen the requirement of physical interaction of hCdc73 with FMR1 for the recruitment of hCdc73 to SGs. In addition, a GST pull-down assay employing hCdc73 protein missing IDR(N) will strengthen the importance of this region for FMR1-mediated recruitment to SGs.
9. To strengthen selective translocation of hCdc73 to SGs, it is recommended to check the association of hCdc73, eEF1Bc, and Ski8 (by co-immunoprecipitation or relevant analysis) in the presence and absence of stress signals.
10. Related to the above issue (9), can the authors provide experimental evidence and/or possible mechanism for how stress signals dissociate hCdc73 from the hCdc73/eEF1B&[gamma]/Ski8 complex?

Reviewer 2

Advance summary and potential significance to field

In this manuscript, Lee et al. expand on their 2014 study (Jo, et al. Nature Comm.) investigating the cytoplasmic role of CDC73 in the post-transcriptional regulation of tumor suppressor p53. Here, they report the recruitment of a sub-population of CDC73 protein to stress granules (SG), phase-separated structures that form in response to various stress treatments. Using deletion mapping the authors identify a putative phase separation/targeting domain(s) and propose a recruitment mechanism that requires the SG resident FMR1. Lee and co-workers also propose that this SG recruitment facilitates an additional layer of CDC73-mediated regulation for p53 mRNA, whereby the CDC73/Ski8/eEF1Bg-associated degradation of this transcript is blocked by sequestration of CDC73 during stress. While there are some interesting observations presented within this manuscript, this Reviewer has concerns about key elements of the work.

© 2023. Published by The Company of Biologists under the terms of the Creative Commons Attribution License (https://creativecommons.org/licenses/by/4.0/).

Comments for the author

a) Only an extremely small sub-population of CDC73 appears to be targeted to SGs. Even if the nuclear population is excluded (the majority of the protein), there stills seems to be a large pool of free-cytosolic CDC73 (endogenous) during the various stress treatments. Therefore, it is very difficult to envision how the recruitment of only a small sub-population of CDC73 to SGs could mediate the mechanism proposed by the authors. SGs and CDC73 may play a role in the stress-induced stabilization of p53 mRNA, but the data demonstrating that it is "…controlled by CDC73 sequestration to stress granules" (as described in the manuscript title) is not convincing.
b) The heat shock data doesn't appear to add to the story presented by the authors. Also, 46oC is an extremely harsh treatment temperature, which seems out of line with some of the common practices in the literature. 42oC (Gilks et al, MBoC, 2004) or 43oC (Sama et al, J Cell Physiol, 2013) seem to be more commonly used to induce SG formation. To use this extreme temperature, the authors would need to (1) justify why a more traditional temperature has not been selected and (2) demonstrate that the cells remain viable during the stress exposure.
c) Similar to the point above, an 8 hr (2hr + 6hr maintenance) and 14 hr (2hr + 12hr maintenance) sodium arsenite treatment seems extreme. The viability of the cells should be demonstrated for these harsh treatment conditions.
d) There are issues with the mapping of the SG targeting motif/IDR data (Figures 2-4):
a. In Figure 2 the SG enrichment score of the control seems to be highly variable. Figure 2C has a value of ~15 for NLSmut, while the same construct in Figure 2E has a score of ~32. It is unclear why this would have such high variability. It is also unclear how many independent data sets were used to calculate these values.
b. The interpretation of the mapping data (Figures 2-4) is unclear and seems unfinished. The authors propose that IDR(C) imparts phase separation properties, while IDR(N) mediates FMR1 binding (i.e., SG targeting). However, the data doesn't seem to fully agree with the interpretation. In Figure 2D/E, why would IDR(N)-targeting mirror the NLSmut if it is incapable of phase separation? Also, why wasn't the in vitro droplet formation assay performed with IDR(N) and IDR(C) to further strengthen this argument (in theory only IDR(C) should separate)?
c. It's unclear how the siRNA experiments in Figure 4A-C were performed. Two different siRNA constructs are listed for each SG resident (Supplementary Table). Were the siRNAs used independently to validate each other, or pooled to potentially increase the knockdown? Were rescue experiments performed, expressing exogenous FMR1, to confirm the specificity of the effect?
d. Figure 4D is poorly laid out. Why is the loading order markedly different between the input and GST pulldown? Was this experiment only performed once?
e. The data in Figure 4D needs to be fleshed out to strengthen the results and bring the story in figures 2-4 together. Including GST-IDR(C) and deltaIDR(N) would demonstrate that FMR1 binding to IDR(N) is specific, since this figure has no region of CDC73 used as a negative control (GST alone also seems to be missing). Other proteins from Figure 4A could also be tested to demonstrate that IDR(N)/full-length CDC73 are not randomly associating with SG proteins in vitro.
f. Figure 1E and 1F seem out of place in Figure 1. They might be better situated in Figure 4. Additionally, why is endogenous CDC73 knocked-down in the siCtrl-treated sample of panel Figure 1E?
g. The authors state, "… we conclude that FMR1, as a carrier protein, brings hCDC73 to SGs via physical interactions." FMR1 inhibition only leads to about a ~ 50% reduction in SG targeting and there are potential issues with the in vitro interaction data (described above). Thus, this seems to be a too strong/definitive statement, which isn't supported by enough data. Additional experiments would be needed to make this claim.
e) For Figure 5A, the authors state, "We observed statistically significant changes in p53 mRNA levels, except under TG-induced stress conditions." Based on the model proposed by the authors this doesn't seem to make sense. Figure 1B suggests that targeting of CDC73 to SG occurs at a similar level in ARS, MG132, and TG conditions, yet there is no stabilization of p53 mRNA under TG conditions. How is this reconciled with the model?
f) To believe the interpretation of the data in Figure 5J we need to assume that the rescue construct CDC73-deltaIDR(N+C) is functionally active. Is there any evidence that this construct is active? The authors should be able to test this, since they wrote the paper describing the cytoplasmic role of CDC73 (Jo, et al. Nature Comm.).
g) Figure 5G and 5I suggest that CDC73 and SG formation play a role in p53 mRNA stability during stress treatment. However, there is no convincing data that CDC73 targeting to SG is necessary for

© 2023. Published by The Company of Biologists under the terms of the Creative Commons Attribution License (https://creativecommons.org/licenses/by/4.0/).

this effect. Creating a system where a functional version of CDC73 is excluded from the SG would be necessary to make this connection. Perhaps the authors could assess the effects of FMR1 knockdown on p53 stability. While this is a potentially imperfect solution (FMR1 could directly regulate p53), by the authors account it impairs endogenous CDC73 recruitment, which should reduce p53 mRNA stability during stress treatment.
h) The authors use a lack of co-localization of p53 mRNA, CDC73, and SGs to validate their model of CDC73 disassociation. However, in this manuscript they never show p53 mRNA/CDC73 co-location under untreated conditions, as a control for the association. This makes the data presented here less compelling.
i) The data in Figure 6D would be more convincing if NLSmut and deltaIDR(N+C) were included as controls in this experiment.
j) As a minor point, the layout of the figures is somewhat frustrating. Some specific comments are below:
a. The CDC73 images seem to switch between the second (1D and 1E) and third (1B and 1I) columns of the Figure 1 panels. They should be consistent throughout the manuscript.
b. The amino acid substitutions of the NLSmut construct contain typos in both Figure 1C and 2A. What does R126 and 139A (Figure 1C) and R126 and 13 (Figure 2A) mean?
c. Several panels should be transferred to supplementary information. The validation of siRNA knockdowns (Figure 1F-left, 4B, and 5I-left) and schematics of the culture conditions (Figure 1G and 5D) are strong candidates for movement to supplemental material.
Overall, there are elements of this work that could be of interest to the Journal of Cell Science readership, however, the story appears to be incomplete at this time. It is unclear whether the data adequately addresses the primary hypothesis that CDC73 sequestration in stress granules controls p53 availability, so this reviewer can't recommend this work for publication.

<u>Reviewer 3</u>

Advance summary and potential significance to field

hCdc73 is a component of exosome complex and was previously found to regulate p53 stability. Herein, the authors examined a possible mechanism for this regulation. It is shown that hCdc73 is recruited into stress granules (SG) upon stress, and sequestration is induced by previously unidentified disordered region within cdc73. In vitro and an in vivo model system support this observation. Moreover siRNA to Fmr1 indicated a role in localization of cdc73 to SG. Mechanistically the authors observed association of cdc73 with p53 mRNA through a bridging protein. siRNA to cdc73 resulted in an increase in p53 in a manner that is independent of its transcription. Thus, sequestration of cdc73 to SG, through its predicted DR, is important for posttranscriptional regulation of p53 mRNA. Overall, this work reveal new mechanism for post-transcriptional regulation of mRNA translation, namely though sequestration and release of an mRNA regulators from SG. This work has therefore important implications to our understanding of the roles of SG and their regulatory potential.

Comments for the author
Comments:
1. It is not clear if the disappearance of hCdc73 from SG upon their disappearance (Fig 1H) is due to its dispersal in the cytoplasm or degradation. The authors should do Western blot at this time point to reveal whether cdc73 levels do not change (i.e. it dissociates back to the cytosol) or decrease (i.e. it is degraded).
2. Alphafold 2 predctions suggests clear structures to some of the IDR regions predicted here (https://alphafold.ebi.ac.uk/entry/A0A1B0GUB2) . Can the authors explain the source of discrepancy?
3. The experiment in Fig 3 is missing important negative controls- proteins that do not contain predicted IDR and do not aggregate under these experimental conditions. Most suitable is cdc73deltaIDR(N+C). Furthermore, aggregation of the full length cdc73 should be presented.
4. Fig 5: mRNA quantification is missing control mRNAs. In particular in 5E, to determine if the observed changes upon DRB treatment are specific to targets of cdc73.

© 2023. Published by The Company of Biologists under the terms of the Creative Commons Attribution License (https://creativecommons.org/licenses/by/4.0/).

First revision

Author response to reviewers' comments

Dear Editor,

Thank you for giving us the opportunity to submit a revised manuscript titled "Translocation of cytosolic hCdc73 to stress granules plays a role in the arsenite-induced stabilization of p53 mRNA (modified title from previous version)" to *Journal of Cell Science*. We appreciate the time and effort that you and the reviewers have dedicated to providing your valuable comments on our manuscript. We have been able to incorporate changes to reflect all the suggestions provided by the reviewers. We hope that our revised manuscript is satisfactory, as we have tried our best to address the issues raised by the reviewers. Please see our point-by-point responses to the reviewers' comments below. Each answer is marked in red and indicated with an arrow before the next comment.

Answers to Reviewer 1.

In this manuscript, the authors demonstrate a posttranslational control of p53 mRNA levels mediated by selective sequestration of hCdc73 to stress granules under stress conditions. This work further details the authors' previous finding that showed hCdc73/eEF1B&[gamma]/Ski8 complex-mediated p53 mRNA level control in the cytosol and provides a novel pathway of p53 mRNA level regulation by SGs. Overall, experiments are well designed, conducted, and supportive of conclusions.

This reviewer raises the following issues.

1. Throughout the manuscript, [Paf1 complex, PAF complex, PAFc], [RNAPII, RNA Pol II], and [hCdc73, Cdc73] are used as a mix. It would be good to unify terminology.
→ As the reviewer pointed out, we unified the terminology listed in the comment. "Paf1 complex" and "PAF complex" have been unified to PAFc, and "RNA Pol II" has been changed to "RNAPII". Except for yeast Cdc73, Cdc73 from humans has been unified as "hCdc73".

2. G3BP1 is stained green in most experiments. However, in some figures where G3BP1 is stained pale blue–green (e.g., Fig. 1E, 4A), it is hard to evaluate the colocalization of G3BP1 and Cdc73.
→ As the reviewer suggested, we modified the color of confocal data of G3BP1 to green.

3. In Fig. S2, the figure legend for (top) does not match the top figure. Additionally, a more detailed description for the middle panel is necessary.
→ We apologize for the mistakes. Figure legend for Fig. S2 properly corrected. We also simplified the diagram to deliver the information more clearly. A more detailed description for the middle panel was also added.

4. What is TIA-1 used in Figure 2? The usage of this reagent is not described in Materials and Methods. Are TIAR and TIA-1 same reagent?
→ TIA-1 and TIAR (TIA-1-related protein) are two different marker proteins of stress granules. We added a proper explanation for each marker in the "Results" and 'Materials and Methods" sections. (Results section, page 7).

5. Inclusion of SDS–PAGE/Coomassie Blue staining of purified 6X His-GFP-tagged hCdc73-IDR(N+C) protein as a Supplemental Figure is recommended to demonstrate the quality of purified protein.
→ We added the Coomassie blue staining result of the purified 6X His-GFP-tagged-IDR(N+C) of hCdc73 in Fig S2C.

6. In Fig. 3A and 3B, indicate what DIC means in the figure legend. Is it a Differential Interference Contrast?
→ Yes. We modified the figure legends to indicate that DIC is an abbreviation for differential interference contrast.

7. In Fig 3, in addition to hCdc73 protein fragments, it is more interesting to test whether full-length hCdc73 has a phase separation property because endogenous hCdc73 is present as a full-

© 2023. Published by The Company of Biologists under the terms of the Creative Commons Attribution License (https://creativecommons.org/licenses/by/4.0/).

length form in cells. In addition, an experiment with the hCdc73 protein missing IDR(C) will provide a conclusive answer for the requirement of this region for phase separation.
→ To address the reviewer's comment, we first attempted to purify the full-length form of the Cdc73 protein in vitro. However, it was too large to purify in our experimental setting. To circumvent this problem, we performed an optoDroplet assay with the full-length form of hCdc73^{NLSmut}-mCherry-CRY2. Within the intensity and period of light exposure used for IDR(N+C)-mCherry-CRY2, however, light-activated droplet formation of hCdc73^{NLSmut}-mCherry-CRY2 was not detected. Since the full-length form of hCdc73 did not possess assembly properties by itself, we did not perform optoDroplet assay with hCdc73^{NLSmut}- ΔIDR(C)-mCherry-CRY2. Data presented in Fig. S2D (in the Results section, pg. 9).
→ It is not surprising to observe that the full-length form of the protein does not undergo phase separation, although part of the internal domain (usually IDR) possesses this property (as exemplified in Zhang et al., 2019). DOI: 10.7554/eLife.39578).
→ We added a paragraph postulating the role of hCdc73 (IDR) in the regulation of phase separation in the revised manuscript (in the Results section, page 8, 9).

8. In Fig. 4, the inclusion of GST pull-down assays with (one or two) proteins that did not change the colocalization patterns (e.g., CAPRIN1, FXR1, NUFOP2, UBAP2L, or USP10) will strengthen the requirement of physical interaction of hCdc73 with FMR1 for the recruitment of hCdc73 to SGs. In addition, a GST pull-down assay employing hCdc73 protein lacking IDR(N) will strengthen the importance of this region for FMR1-mediated recruitment to SGs.
→ To address the reviewer's comments, we performed a GST pull-down assay with hCdc73 and various SG scaffold proteins. The results showed that hCdc73 also interacted with CAPRIN1, a known binding partner of FMR1 (Wu et al. 2016). In contrast, interaction with USP10 was not detected. New data and interpretations have been added to the Results section, and the data are presented in Fig. S3D.
→ To address the reviewer's comments, we also performed a GST pull-down assay using hCdc73 protein lacking IDR(N). As expected, the interaction between hCdc73 and FMR1 was diminished when IDR(N) was deleted. These results suggest that hCdc73 binds with FMR1 through the IDR (N) domain, which is crucial for its localization into stress granules. New data are presented in Fig. 4E-F. The Results section was revised accordingly (Results section, page 10).

9. To strengthen the selective translocation of hCdc73 to SGs, it is recommended to check the association of hCdc73, eEF1Bc, and Ski8 (by coimmunoprecipitation or relevant analysis) in the presence and absence of stress signals.
→ To address the reviewer's comment, we tested the association of hCdc73 and eEF1bγ under normal and stress granule-induced conditions. Since eEF1bγ is the key component of the cytoplasmic hCdc73 complex that directly binds to p53 mRNA (Jay-Hyun Jo, et al. 2014), we focused on the interaction between hCdc73 and eEF1bγ.
→ We first monitored the localization of hCdc73 and eEF1bγ under arsenic stress conditions. Unlike hCdc73, the distribution patterns of eEF1bγ did not change after arsenic treatment. As a result, its colocalization within puncta structures (along with hCdc73) under arsenic stress conditions was not observed (Fig. S5C).
→▫We performed coimmunoprecipitation assays multiple times, with different settings, either with tagged exogenous protein or with endogenous proteins. In each case, we repeatedly observed that the physical interaction between hCdc73 and eEF1Bγ was slightly reduced under sodium arsenite treatment conditions (Fig. S5D).
→ We wished to observe a more dramatic reduction. However, considering the amount of cytosolic hCdc73 translocated to SGs, it was as good as we obtained. Only small portions of cytosolic hCdc73 translocate to SGs, and a large amount of cytosolic hCdc73 remains outside of SGs, which supports its interaction with eEF1bγ. We believe that the reduced amount of physical interaction between hCdc73 and eEF1Bγ represents portions of cytosolic hCdc73 translocation to SGs.
→ Data are now presented in Fig. S5. The Results section was revised accordingly (see page 12). In addition, since it was obvious that not all but small portions of cytosolic hCdc73 translocate to SGs, we modified our manuscript thoroughly to point out the concept.

10. Related to the above issue (9), can the authors provide experimental evidence and/or possible mechanism for how stress signals dissociate hCdc73 from the hCdc73/eEF1B&[gamma]/Ski8 complex?

© 2023. Published by The Company of Biologists under the terms of the Creative Commons Attribution License (https://creativecommons.org/licenses/by/4.0/).

→ At this moment, we do not have experimental data explaining how hCdc73 dissociates from the hCdc73/eEF1B/Ski8 complex under SG conditions. However, based on the following two observations (facts), we speculate a possible mechanism based on competition and spatial sequestration as follows.
→ First, the interaction domain of hCdc73 for eEF1B and FMR1 overlaps. In a previous report demonstrating the interaction between hCdc73 and eEF1Bγ, we showed that aa 110-343 of hCdc73 is required for its interaction with eEF1Bγ. In this manuscript, we also demonstrated that the IDR(N) region (256-360 aa) of hCdc73 is needed for its interaction with FMR1. Since those regions for interaction with eEF1Bγ overlap with those of FMR1, it is highly likely that their interaction with hCdc73 is competitive.
→ Second, FMR1, but not eEF1Bγ, can condense under stress conditions. Under basal conditions, FMR1 and eEF1B proteins are localized throughout the cytoplasm; hence, we can speculate that the probability of dispersed hCdc73 protein encountering either FMR1 or eEF1B in the cytosol is similar. In contrast, under stress conditions, cytosolic hCdc73 bound with FMR1 selectively sequestered within SGs; hence, the probability of hCdc73 encountering eEF1B outside of SGs decreased.
→ In a scenario of random association of hCdc73 with competitive eEF1Bγ or FMR1, sequestration of hCdc73 along with FMR1 into restricted cellular compartments results in a lowered probability of eEF1Bγ (and p53 mRNA) association with hCdc73, which we observed.
→ We added a paragraph explaining the possible mechanism in the Discussion section (see page 14).

<u>**Answers to Reviewer 2.**</u>
In this manuscript, Lee et al. expand on their 2014 study (Jo, et al. Nature Comm.) investigating the cytoplasmic role of CDC73 in the posttranscriptional regulation of tumor suppressor p53. Here, they report the recruitment of a subpopulation of CDC73 protein to stress granules (SG), phase-separated structures that form in response to various stress treatments. Using deletion mapping the authors identify a putative phase separation/targeting domain(s) and propose a recruitment mechanism that requires the SG resident FMR1. Lee and coworkers also proposed that SG recruitment facilitates an additional layer of CDC73-mediated regulation of p53 mRNA, whereby the CDC73/Ski8/eEF1Bg-associated degradation of this transcript is blocked by sequestration of CDC73 during stress. While there are some interesting observations presented within this manuscript, this Reviewer has concerns about key elements of the work.

a) Only an extremely small subpopulation of CDC73 appears to be targeted to SGs. Even if the nuclear population is excluded (the majority of the protein), there still seems to be a large pool of free-cytosolic CDC73 (endogenous) during the various stress treatments. Therefore, it is very difficult to envision how the recruitment of only a small subpopulation of CDC73 to SGs could mediate the mechanism proposed by the authors. SGs and CDC73 may play a role in the stress-induced stabilization of p53 mRNA, but the data demonstrating that it is "…controlled by CDC73 sequestration to stress granules" (as described in the manuscript title) is not convincing.
→ Although our original intention was to emphasize "translocation into SG" and "p53 mRNA control", we realized the title was too strong and comprehensive. As the reviewer correctly pointed out, only portions of cytosolic hCdc73 translocate to SGs; hence, portions of p53 mRNA are affected by this event. Therefore, we changed our title from 'p53 mRNA availability during the cellular stress response is controlled by hCdc73 sequestration to stress granules' to "Translocation of hCdc73 to stress granules plays a role in the arsenite-induced stabilization of p53 mRNA". In addition, we changed the manuscript thoroughly to reflect the reviewer's concerns.

b) The heat shock data doesn't appear to add to the story presented by the authors. Additionally, 46˚C is an extremely harsh treatment temperature, which seems out of line with some of the common practices in the literature. 42˚C (Gilks et al, MBoC, 2004) or 43˚C (Sama et al, J Cell Physiol, 2013) seem to be more commonly used to induce SG formation. To use this extreme temperature, the authors would need to (1) justify why a more traditional temperature has not been selected and (2) demonstrate that the cells remain viable during the stress exposure.
→ We repeated our heat shock experiment at 43˚C. Under this condition, we observed similar patterns of hCdc73 translocation to SGs. (New data presented in Fig. 1B)

c) Similar to the point above, an 8 hr (2 hr + 6 hr maintenance) and 14 hr (2 hr + 12 hr maintenance) sodium arsenite treatment seems extreme. The viability of the cells should be demonstrated for these harsh treatment conditions.

© 2023. Published by The Company of Biologists under the terms of the Creative Commons Attribution License (https://creativecommons.org/licenses/by/4.0/).

→ To address the reviewer's concern, we tested the viability of HeLa cells by trypan blue staining under arsenic treatment conditions. The viability of cells treated with sodium arsenite (150 μM) for 8 hours was similar to the viability of cells treated for 2 hours. In contrast, after 14 hours of prolonged treatment, the viability of the cells decreased. However, as demonstrated in Fig. S1I, the morphology of attached cells after 14 hours of treatment was not severely altered. We described the viability and morphology of cells under harsh treatment conditions in the Results section and viability data presented in Fig. S1I (See page 7).

d) There are issues with the mapping of the SG targeting motif/IDR data (Figures 2-4):
d-1. In Figure 2 the SG enrichment score of the control seems to be highly variable. Figure 2C has a value of ~15 for NLSmut, while the same construct in Figure 2E has a score of ~32. It is unclear why this would have such high variability. It is also unclear how many independent data sets were used to calculate these values.
→ We would like to thank the reviewer for pointing out this issue. This is because we employed two different kinds of confocal microscopy, a FLUOVIEW FV3000 confocal microscope (Olympus, Tokyo, Japan) and a TCS SP5 confocal microscope (Leica, Wetzlar, Germany). As a result, in addition to image acquisition, image processing was executed utilizing different software. We postulate that those differences might cause the variance in the SG enrichment score.
→ Based on the reviewer's comment, we reperformed the IDR-only mutant experiment using the 'TCS SP5 confocal microscope'. The new image and its quantitation result are presented in Fig 2D and 2E.
→ Three biologically independent experiments were conducted to perform statistical analyses. We analyzed more than 10 cells by random selection among the three independent data sets. Data are presented in the revised Fig. 2 and stated in the 'Materials and method' section.

d-2. The interpretation of the mapping data (Figures 2-4) is unclear and seems unfinished. The authors propose that IDR(C) imparts phase separation properties, while IDR(N) mediates FMR1 binding (i.e., SG targeting). However, the data do not seem to fully agree with the interpretation. In Figure 2D/E, why would IDR(N)-targeting mirror the NLSmut if it is incapable of phase separation? Additionally, why wasn't the in vitro droplet formation assay performed with IDR(N) and IDR(C) to further strengthen this argument (in theory only IDR(C) should separate)?
→ We admit that our original data set was somewhat unclear and unfinished. Most of the confusion comes from the role of IDR(C), i.e., whether it is sufficient to impart phase separation properties by itself. To address this question, we performed an in vitro droplet assay using purified IDR(C) and IDR(N) proteins. The data clearly showed that IDR(C) by itself does not form droplets in vitro. (it was more aggregated form). The data are presented in Fig. 3C.
→ Since IDR(N+C) forms droplets in vitro, these data indicate that molecular interactions between IDR(C)-IDR(C) might be too strong to phase separate, and the presence of IDR(N) somewhat loosens the strength between IDR(C)-IDR(C), low enough to undergo phase separation. We added a more detailed explanation in the Results section on pages 8 and 9.
→ Truncated IDR(N) alone was sufficient to translocate to SGs, probably via interaction with FMR1. However, the full-length form of hCdc73 without the IDR-C domain did not translocate to SGs, indicating that an as-yet-unidentified domain in hCdc73 blocks its uncontrolled translocation into SGs.
→ We added a more detailed explanation of our working model in the Discussion (see page 14).

d-3. It's unclear how the siRNA experiments in Figure 4A-C were performed. Two different siRNA constructs are listed for each SG resident (Supplementary Table). Were the siRNAs used independently to validate each other, or pooled to potentially increase the knockdown? Were rescue experiments performed, expressing exogenous FMR1, to confirm the specificity of the effect?
→ We transfected pooled siRNA to increase the knockdown efficiency. We revised the 'Material and method' section accordingly.
→ To address the reviewer's comments, we performed a rescue experiment expressing exogenous GFP-FMR1. To clarify our experimental conditions, we first knocked down FMR1 using siRNA and verified that SG enrichment of hCdc73 was decreased (Fig. 4C). Then, we reconstituted cells with GFP-FMR1, which rescued the SG localization of hCdc73 (Fig. 4C, D). We added new images and quantitated data in Fig. 4C, D.

© 2023. Published by The Company of Biologists under the terms of the Creative Commons Attribution License (https://creativecommons.org/licenses/by/4.0/).

d-4. Figure 4D is poorly laid out. Why is the loading order markedly different between the input and GST pulldown? Was this experiment only performed once?
→ We apologize for the confusion. It was our mistake in the step of protein loading. We did not pay much attention earlier. Pulldown experiments were performed several times with similar results. Furthermore, we repeatedly used the FMR and hCdc73 interaction as a positive control for our further experiments (i.e., domain mapping or interaction with other SG carrier proteins). To avoid confusion, however, we changed the presented data set with the proper loading order (Fig. 4E).

d-5. The data in Figure 4D needs to be fleshed out to strengthen the results and bring the story in figures 2-4 together. Including GST-IDR(C) and deltaIDR(N) would demonstrate that FMR1 binding to IDR(N) is specific, since this figure has no region of CDC73 used as a negative control (GST alone also seems to be missing). Other proteins from Figure 4A could also be tested to demonstrate that IDR(N)/full-length CDC73 is not randomly associated with SG proteins in vitro.
→ As the reviewer suggested, we performed a GST pull-down assay with GST alone, GST- ΔIDR (N), along with GST-hCdc73, to test whether the binding with FMR1 is IDR (N) specific. The data show that the IDR (N) region is required for the interaction between hCdc73 and FMR1. New data are presented in Fig. 4F.
→ Separately, we tested other SG-scaffold proteins for interaction with hCdc73. Among the tested, CAPRIN1, a known binding partner of FMR1, interacts with hCdc73. In contrast, USP10 and G3BP1 did not interact with hCdc73, suggesting that IDR(N)/full-length hCDC73 is not randomly associated with SG scaffold proteins. (Fig. S3D). (Result section. Page 10)

d-6. Figure 1E and 1F seem out of place in Figure 1. They might be better situated in Figure 4. Additionally, why is endogenous CDC73 knocked-down in the siCtrl-treated sample of panel Figure 1E?
→ When we observed translocation of hCdc73 into SGs in Fig. 1A-D, there were two questions. The first one was "how?" The second one was "why?" Before we moved into the mechanism of translocation, we wanted to rule out the possibility of hCdc73 controlling the SG assembly processes itself. This was the main reason we positioned the hCdc73 siRNA data in Fig. 1. We added a sentence explaining the purpose of this experiment in the Results section of Fig 1E.
→ In Fig 1E, endogenous hCdc73 is not knocked-down in the siCtrl-treated sample. I wonder whether the display of images causes some confusion. We changed the data more clearly so that siCtrl and siCdc73 images showed distinct differences in their hCdc73 expression.

d-7. The authors state, "… we conclude that FMR1, as a carrier protein, brings hCDC73 to SGs via physical interactions." FMR1 inhibition only leads to about an ~ 50% reduction in SG targeting and there are potential issues with the in vitro interaction data (described above). Thus, this seems to be a too strong/definitive statement, which isn't supported by enough data. Additional experiments would be needed to make this claim.
→ Based on the experiments we performed during the revision process, we agree that our original claim was too strong and definitive. As the reviewer correctly pointed out, only portions of cytosolic hCdc73 are subject to translocation into SGs. Therefore, we changed sentences of "… we conclude that FMR1, as a carrier protein, brings hCDC73 to SGs via physical interactions" to "FMR1 helps the translocation of hCdc73 to SGs via physical interaction with the IDR(N)." in the revised manuscript. In addition, we checked our manuscript thoroughly and tone-down the claim.

e) For Figure 5A, the authors state, "We observed statistically significant changes in p53 mRNA levels, except under TG-induced stress conditions." Based on the model proposed by the authors this doesn't seem to make sense. Figure 1B suggests that targeting of CDC73 to SG occurs at a similar level in ARS, MG132, and TG conditions, yet there is no stabilization of p53 mRNA under TG conditions. How is this reconciled with the model?
→ Under arsenic stress conditions, we claim that changes in p53 mRNA levels are partly controlled by SG translocation of cytosolic hCdc73. We are confident that our data support this claim under sodium arsenite-treated conditions. We also understand that we cannot generalize this claim to all stress conditions. Upon reading the reviewer's comments, we realized that our statement (including the title) was too strong to mislead this generalization.
→ As the reviewer correctly pointed out, p53 level changes were not detected under TG stress conditions, although hCdc73 translocated to SGs. We repeated the experiment several times under different conditions, but p53 level changes were not detected under TG stress conditions, although hCdc73 translocation occurred. In contrast, p53 level changes were well detected under MG132

© 2023. Published by The Company of Biologists under the terms of the Creative Commons Attribution License (https://creativecommons.org/licenses/by/4.0/).

stress conditions, which correlates with hCdc73 translocation into SGs. There are many hypotheses to test this result, yet, I am afraid it is beyond our current scope.
→ To avoid overstatement and generalization, we changed our title from 'p53 mRNA availability during the cellular stress response is controlled by hCdc73 sequestration to stress granules' to "Translocation of cytosolic hCdc73 to stress granules plays a role in the arsenite-induced stabilization of p53 mRNA". We modified the whole manuscript to tone down the overstatement and to avoid generalization.
→ Nonetheless, we wish to keep TG- and MG132-stress data in Fig 5 because we believe this result is worth sharing. Instead, we clearly stated that it is a simple description, not a generalization, and our claim is valid for sodium arsenite and MG132 induced conditions. New data are presented in Fig. S4D-F.

f) To support the interpretation of the data in Figure 5J, we need to assume that the rescue construct CDC73-deltaIDR(N+C) is functionally active. Is there any evidence that this construct is active? The authors should be able to test this, since they wrote the paper describing the cytoplasmic role of CDC73 (Jo, et al. Nature Comm.
→ As the reviewer suggested, we confirmed whether hCdc73^{NLSmut}-ΔIDR(N+C) is functionally active, similar to hCdc73^{NLSmut}, in p53 mRNA destabilization (Jo et al., 2014). Although the interaction of hCdc73^{NLSmut}-ΔIDR(N+C) with p53 mRNA was somewhat decreased compared to that of hCdc73^{NLSmut}, it was enough to destabilize p53 mRNA. These data are now presented in Fig. S4L and Fig. 6F.

g) Figure 5G and 5I suggest that CDC73 and SG formation play a role in p53 mRNA stability during stress treatment. However, there are no convincing data that CDC73 targeting to SGs is necessary for this effect. Creating a system where a functional version of CDC73 is excluded from the SG would be necessary to make this connection. Perhaps the authors could assess the effects of FMR1 knockdown on p53 stability. While this is a potentially imperfect solution (FMR1 could directly regulate p53), by the authors account it impairs endogenous CDC73 recruitment, which should reduce p53 mRNA stability during stress treatment.
→ We tried to create a system to address the reviewer's concern, but we could only come up with the idea that the 'functional version of hCdc73 excluded from the SG' was hCdc73^{NLSmut}-ΔIDR(N+C) . Because the reviewer previously commented, we knew the functional activity of hCdc73^{NLSmut}-ΔIDR(N+C) was normal to that of hCdc73^{NLSmut} (Fig S4L).
→ Furthermore, binding between hCdc73^{NLSmut}-ΔIDR(N+C) and p53 mRNA was not decreased, rather increased under SG-inducing conditions in comparison with hCdc73^{NLSmut}(Fig. 6F).
→ We also silenced hCdc73 and then reconstituted it with hCdc73^{NLSmut} or hCdc73^{NLSmut}-ΔIDR(N+C). Reconstitution with hCdc73^{NLSmut} after the knockdown of hCdc73 restored SG-induced p53 mRNA upregulation. However, there was no detectable restoration in cells reconstituted with the IDR(N+C) deletion mutant (Fig. 5I).
→ These data collectively imply that hCdc73 targeting to SG through its IDR domain is necessary for p53 mRNA stabilization when stressed with sodium arsenite.
h) The authors use a lack of colocalization of p53 mRNA, CDC73, and SGs to validate their model of CDC73 disassociation. However, in this manuscript they never show p53 mRNA/CDC73 colocation under untreated conditions, as a control for the association. This makes the data presented here less compelling.
→ As the reviewer requested, we performed FISH to monitor the colocalization of hCdc73 protein and p53 mRNA. Unfortunately, however, staining conditions for endogenous hCdc73 antibodies do not work under FISH conditions.
→ In FISH, cells are fixed in methanol. In this condition, hCdc73 protein staining does not reflect its normal staining pattern (compared to paraformaldehyde fixation, see below Data#1). Nonetheless, we stained cells for hCdc73 protein and p53 mRNA, along with G3BP1 (see below Data #2). As expected, good amounts of p53 mRNA were codetected, along with broadly distributed hCdc73 protein.

i) The data in Figure 6D would be more convincing if NLSmut and deltaIDR(N+C) were included as controls in this experiment.
→As the reviewer suggested, we performed RNA-IP with both hCdc73^{NLSmut} and hCdc73^{NLSmut}-ΔIDR(N+C) and monitored the change in p53 mRNA binding fold change when stressed with sodium arsenite to induce SG. The results showed that the hCdc73^{NLSmut}-interacting p53 mRNA level decreased 0.7-fold under SG-inducing conditions. On the other hand, hCdc73^{NLSmut}-ΔIDR(N+C)-interacting p53 mRNA increased 1.5-fold. We interpreted these differences to be derived from the

© 2023. Published by The Company of Biologists under the terms of the Creative Commons Attribution License (https://creativecommons.org/licenses/by/4.0/).

translocation of hCdc73^{NLSmut} to SGs, while the IDR(N+C)-deleted form does not. The new data are presented in Fig. 6F.

j) As a minor point, the layout of the figures is somewhat frustrating. Some specific comments are below:
j-a. The CDC73 images seem to switch between the second (1D and 1E) and third (1B and 1I) columns of Figure 1 panels. They should be consistent throughout the manuscript.
→ As commented, we arranged the columns of Cdc73 and G3BP1 in a consistent order.

j-b. The amino acid substitutions of the NLSmut construct contain typos in both Figure 1C and 2A. What does R126 and 139A (Figure 1C) and R126 and 13 (Figure 2A) mean?
→ We apologize for these mistakes. Those typos are fixed.

j-c. Several panels should be transferred to supplementary information. The validation of siRNA knockdowns (Figure 1F-left, 4B, and 5I-left) and schematics of the culture conditions (Figure 1G and 5D) are strong candidates for movement to supplemental material.
→ As the reviewer suggested, we moved those panels to the Supplementary section, except Figure 1G. We think the first schematics of the sodium aresenite treatment (Fig 1G) helps the readers to understand the concept of SG-maintenance and disassembly condition in our experiments.

Overall, there are elements of this work that could be of interest to the Journal of Cell Science readership, however, the story appears to be incomplete at this time. It is unclear whether the data adequately addresses the primary hypothesis that CDC73 sequestration in stress granules controls p53 availability, so this reviewer can’t recommend this work for publication.

Answers to Reviewer 3.

hCdc73 is a component of the exosome complex and was previously found to regulate p53 stability. Herein, the authors examined a possible mechanism for this regulation. hCdc73 is recruited into stress granules (SGs) upon stress, and sequestration is induced by a previously unidentified disordered region within cdc73. In vitro and in vivo model systems support this observation. Moreover, siRNA to Fmr1 indicated a role in the localization of cdc73 to SGs. Mechanistically, the authors observed association of cdc73 with p53 mRNA through a bridging protein. siRNA against cdc73 resulted in an increase in p53 in a manner that was independent of its transcription. Thus, sequestration of cdc73 to SGs, through its predicted DR, is important for posttranscriptional regulation of p53 mRNA. Overall, this work reveals a new mechanism for the posttranscriptional regulation of mRNA translation, namely, the sequestration and release of mRNA regulators from SGs. This work therefore has important implications for our understanding of the roles of SGs and their regulatory potential.

Comments:
1. It is not clear if the disappearance of hCdc73 from SG upon their disappearance (Fig 1H) is due to its dispersal in the cytoplasm or degradation. The authors should do Western blot at this time point to reveal whether cdc73 levels do not change (i.e., it dissociates back to the cytosol) or decrease (i.e., it is degraded).
→ We appreciate the reviewer’s comments. We monitored the cytosolic protein level of hCdc73 during disassembly conditions. However, we did not observe severe changes in cytosolic hCdc73 protein levels under these conditions. We postulate that the cytosolic hCdc73 protein likely dissociates back to the cytosol. Data presented in Fig. S1J.

2. Alphafold 2 predctions suggest clear structures to some of the IDR regions predicted here (https://alphafold.ebi.ac.uk/entry/A0A1B0GUB2). Can the authors explain the source of discrepancy?
→ As the reviewer asked, we checked the Alphafold 2 program and found that there are various versions of hCdc73 structure predictions. A0A1B0GUB2, the one reviewer referenced, was based on hCdc73 with a 243-324 aa deletion. In our study, we used the full-length form of hCdc73 (1-531 aa) for IDR prediction.
→ We further monitored the structure of the full-length version of hCdc73 using Alphafold 2. (https://alphafold.ebi.ac.uk/entry/Q6P1J9). This prediction tool clearly shows an unstructured

© 2023. Published by The Company of Biologists under the terms of the Creative Commons Attribution License (https://creativecommons.org/licenses/by/4.0/).

region that matches our predicted IDR domain (256-416 aa). Predicted structure using the Alphafold 2 program presented in Fig. S2B

3. The experiment in Fig 3 is missing important negative controls- proteins that do not contain predicted IDR and do not aggregate under these experimental conditions. The most suitable is cdc73deltaIDR(N+C). Furthermore, aggregation of the full length cdc73 should be presented.
→ To address the reviewer's comment, we first attempted to purify the full-length form of the Cdc73 protein in vitro. However, it was too large to purify in our experimental setting. To circumvent this problem, we next performed an optoDroplet assay with the full-length form of $hCdc73^{NLSmut}$-mCherry-CRY2. Within the intensity and period of light exposure used for IDR(N+C)-mCherry-CRY2, however, light-activated droplet formation of $hCdc73^{NLSmut}$-mCherry-CRY2 was not detected. Since the full-length form of hCdc73 did not possess assembly properties by itself, we did not perform optoDroplet assay with $hCdc73^{NLSmut}$- ΔIDR(C)-mCherry-CRY2. Data presented in Fig. S2D (in the Results section, pg. 9).

4. Fig 5: mRNA quantification is missing control mRNAs. In particular, in 5E, we determined whether the observed changes upon DRB treatment are specific to targets of cdc73.
→ To our understanding, the reviewer was concerned about the level of control mRNAs that are not destabilized by hCdc73 under sodium arsenite-treated conditions (or both sodium arsenite and DRB). Unfortunately, we have not profiled the mRNA groups that are regulated by hCdc73. We admit our limitation that we could not generalize that the regulation when hCdc73 translocates to SGs only applied to target mRNAs of cytosolic hCdc73.
→ To identify control mRNAs, we examined microarray data from our previous research (Jo et al., 2014) to identify mRNAs that are not affected by siCdc73. We selected HRIP3 and Notch1 as putative hCdC73 nontarget mRNAs. We then tested whether sodium arsenite-induced SG formation conditions changed their mRNA levels. The results showed that unlike p53 mRNA, HRIP3 and Notch1 mRNA did not change under arsenic stress conditions (Data#3 below)

Second decision letter

MS ID#: JOCES/2022/260593

MS TITLE: Translocation of cytosolic hCdc73 to stress granules plays a role in arsenic stress-induced stabilization of p53 mRNA

AUTHORS: Hojin Lee, Tae-Hyeon Kim, and Joo Yeon Yoo
ARTICLE TYPE: Research Article

We have now reached a decision on the above manuscript.

To see the reviewers' reports and a copy of this decision letter, please go to: https://submit-jcs.biologists.org and click on the 'Manuscripts with Decisions' queue in the Author Area. (Corresponding author only has access to reviews.)

As you will see, the reviewers recognize that most of their initial criticisms have been addressed in your revised manuscript. However, they still raised issues that will require amendments to your manuscript. I hope that you will be able to carry these out, because I would like to be able to accept your paper.

Please ensure that you clearly highlight all changes made in the revised manuscript. Please avoid using 'Tracked changes' in Word files as these are lost in PDF conversion.

I should be grateful if you would also provide a point-by-point response detailing how you have dealt with the points raised by the reviewers in the 'Response to Reviewers' box. Please attend to all of the reviewers' comments. If you do not agree with any of their criticisms or suggestions please explain clearly why this is so.

© 2023. Published by The Company of Biologists under the terms of the Creative Commons Attribution License (https://creativecommons.org/licenses/by/4.0/).

Reviewer 1

Advance summary and potential significance to field

This manuscript proposes a new mechanism of p53 regulation under stress conditions via stress granules.

Comments for the author

In the revised manuscript, the authors added new data and interpretation to strengthen Cdc73-mediated posttranscriptional regulation of p53 mRNA level under stress conditions. This reviewer raises the following questions and concerns.
1. It was assumed that hCdc73NLSmut-ΔIDR(N+C) is functionally active. However IDR(N+C) (256-360 aa) contains a region required for interaction with eEF1Bγ (110-343 aa). Therefore, hCdc73NLSmut-ΔIDR(N+C) may not interact with eEF1Bγ and thus cannot destabilize p53 mRNA. It is necessary to test whether hCdc73NLSmut-ΔIDR(N+C) interacts with eEF1Bγ.
2. Behavior of IDR(C) is somewhat confusing. This reviewer wonders why strong IDR(C)-IDR(C) interaction that leads to aggregation does not happen in the case of mCherry-hCdc73 IDR(C)-Cry2 protein.
3. Some newly added data need to be appropriately presented. For example,
(1) the order of Fig. S1H~J needs to be reorganized according to the text.
(2) Some explanation for Fig. S1B-D needs to be added in the main text or figure legends. (Why were these experiments done? What do these results mean?)
(3) Unlike the figure legend, NT is not presented in Fig. 2B.

It is suggested to check the text and figure legends throughout the manuscript.

Reviewer 2

Advance summary and potential significance to field

This manuscript explores the targeting of CDC73 to cytoplasmic stress granules and how this regulates p53 mRNA levels/stability. Targeting and phase-separation domains are mapped in CDC73, and an SG resident (FMR1) is described that may mediate recruitment. In the response letter and revised manuscript, the authors have worked to address many of the comments previously provided. They generated new data to strengthen their results, and changed text (particularly the title) to soften their conclusions. This effort has substantially increased the quality of the work, which could be of interest to the JCS community.

Comments for the author

There are four points that were not adequately addressed in the latest submission that need to be clarified before publication can be considered:
1) There are several issues remaining with the siRNA.
a. First, it is not adequately described in the Materials and Methods how the siRNAs were used. The authors mentioned transfecting “pooled siRNA to increase the knockdown efficiency”, but this doesn’t appear in the manuscript. It is unclear how many were transfected for each experiment, as some targets had four (CAPRIN1, USP10), three (FXR, NUFIP2) or only two (UBAP2L, FMR1, CDC73, G3BP1, G3BP2) siRNA listed. It is also unclear when the siRNAs were transfected (i.e., how many hours before treatment), and what transfection reagent was used.
b. While the rescue experiment in Figure 4C could provide some important insight into the mechanism, the methodology of this work is not explained. The authors state, “we first knocked down FMR1 using siRNA and verified that SG enrichment of hCdc73 was decreased (Fig. 4C). Then, we reconstituted cells with GFP-FMR1, which rescued the SG localization of hCdc73 (Fig. 4C, D)” A more precise description needs to be given (and included in the manuscript). Was the siRNA transfected on Day 1, the GFP-FMR1-encoding plasmid transfected 24 hours later, then the stress applied 24 hours after that? Also, how did the plasmid derived FMR1 escape degradation by the

© 2023. Published by The Company of Biologists under the terms of the Creative Commons Attribution License (https://creativecommons.org/licenses/by/4.0/).

siRNA? The siRNA target sequences correspond to amino acids 66-72 (siFMR1#1) and 98-103 (siFMR1#2) of FMR1. These regions would be present in the exogenous construct as well. Often rescue experiments are done using siRNA that target the 5' or 3'-UTR of the mRNA (i.e., regions not found in the plasmid-derived transcript) or by mutating the siRNA binding site of the plasmid derived FMR1 to avoid siRNA binding. More information is required to interpret these results.
c. Thirdly, when analyzing the binding sites for FMR1 (above) this reviewer noted that these siRNAs appear to have been designed to target the mouse, not human FMR1 transcripts. In human HeLa cells, siFMR1#1 might be ok (one nucleotide doesn't align), but the efficiency of siFMR1#2 is extremely questionable (3 nucleotide mismatches). As this is a critical element of the manuscript, it warrants repeating with appropriately designed siRNA.
2) The authors have clarified the number of repeats for the statistical analysis ("We analyzed more than 10 cells by random selection among the three independent data sets"), but the graphs (Figure 2C, 2E, 4B, 4D, and 5I) are still confusing. The legend in Figure 2C/E states, "Quantification of … within SGs (n=11-12)" and there a 11-12 dots/squares/triangles for each sample. What do those dots mean? Are they the results form one cell in one repeat? Based on the authors description, shouldn't there be at least 30 dots (~10 for each of the three repeats)? Further clarification is required here.
3) In Figures 4E, 4F, and S3D it appears that GST alone was added to some of the samples (e.g. lanes 1 and 3 of Figure 4E). This is not described in the figure or the figure legend and needs to be corrected.
4) The authors have not actually addressed the comment, "The amino acid substitutions of the NLSmut construct contain typos in both Figure 1C and 2A. What does R126 and 139A (Figure 1C) and R126 and 13 (Figure 2A) mean?". Looking at the CDC73 sequence it appears that 139A should read R139A, but it's unclear what amino acid R126 was converted to.

Reviewer 3

Advance summary and potential significance to field

I indicated the significance to the field in the previous round of reviewing this work. This significance still holds.

Comments for the author

The authors properly covered all my concerns. I do not have any additional comments.

Second revision

Author response to reviewers' comments

Answ ers to Review er 1.

In the revised manuscript, the authors added new data and interpretation to strengthen Cdc73-mediated posttranscriptional regulation of p53 mRNA level under stress conditions. This reviewer raises the following questions and concerns.

1. It was assumed that hCdc73NLSmut-ΔIDR(N+C) is functionally active. However, IDR(N+C) (256-360 aa) contains a region required for interaction with eEF1Bγ (110-343 aa). Therefore, hCdc73NLSmut-ΔIDR(N+C) may not interact with eEF1Bγ and thus cannot destabilize p53 mRNA. It is necessary to test whether hCdc73NLSmut-ΔIDR(N+C) interacts with eEF1Bγ.

□ To address the reviewer's concern, we checked whether hCdc73^{NLSmut}-ΔIDR(N+C) interacts with eEF1Bγ using an immunoprecipitation assay. A physical interaction between ΔIDR(N+C) and endogenous eEF1Bγ was still detected, indicating that hCdc73^{NLSmut}- ΔIDR(N+C) is functionally

© 2023. Published by The Company of Biologists under the terms of the Creative Commons Attribution License (https://creativecommons.org/licenses/by/4.0/).

active, although it failed to translocate SGs. New data are presented in Fig. S4M. (Results section, page 11).

2. The behavior of IDR(C) is somewhat confusing. This reviewer wonders why strong IDR(C)- IDR(C) interaction that leads to aggregation does not happen in the case of mCherry-hCdc73 IDR(C)-Cry2 protein.

¤ Aggregated behavior of IDR(C) detected *in vitro* (tested with IDR(C) purified protein) and in transfected cells (IDR(C)-myc overexpression). However, as the reviewer correctly pointed out, aggregation did not occur in the case of IDR(C)-mCherry-Cry2. A large difference between these two cases is the size of the fusion protein. In IDR(C)-mCherry-Cry2, IDR(C) is fused with mCherry protein and Cry2 at the C-terminal end. We postulate that the presence of the fusion protein might interfere with the strong interaction between IDR(C). We added this postulation to the manuscript (Results section, page 9).

3. Some newly added data need to be appropriately presented. For example, (1) the order of Fig. S1H~J needs to be reorganized according to the text. (2) Some explanation for Fig. S1B- D needs to be added in the main text or figure legends. (Why were these experiments done? What do these results mean?) (3) Unlike the figure legend, NT is not presented in Fig. 2B. It is suggested to check the text and figure legends throughout the manuscript.

¤ As the reviewer suggested, 1) we reorganized Fig. S1 according to the text. 2) We added an explanation for Fig. S1B-D in the revised manuscript (Results section, page 6). In addition, 3) Figure legends for Fig. 2B changed. We checked the text and figure legends throughout the manuscript. We apologize for the careless mistakes.

<u>**Answers to Reviewer 2.**</u>

This manuscript explores the targeting of CDC73 to cytoplasmic stress granules and how this regulates p53 mRNA levels/stability. Targeting and phase-separation domains are mapped in CDC73, and an SG resident (FMR1) is described that may mediate recruitment. In the response letter and revised manuscript, the authors have worked to address many of the comments previously provided. They generated new data to strengthen their results and changed text (particularly the title) to soften their conclusions. This effort has substantially increased the quality of the work, which could be of interest to the JCS community.

There are four points that were not adequately addressed in the latest submission that need to be clarified before publication can be considered.

1) There are several issues remaining with the siRNA.

a. First, it is not adequately described in the Materials and Methods how the siRNAs were used. The authors mentioned transfecting “pooled siRNA to increase the knockdown efficiency”, but this doesn’t appear in the manuscript. It is unclear how many were transfected for each experiment, as some targets had four (CAPRIthe N1, USP10), three (FXR, NUFIP2) or only two (UBAP2L, FMR1, CDC73, G3Bthe P1, G3BP2) siRNA listed. It is also unclear when the siRNAs were transfected (i.e., how many hours before treatment), and what transfection reagent was used.

¤ To address the reviewer’s concern, we revised the Materials and Methods section to incorporate more detailed information regarding the siRNA transfection procedure. (Materials and methods section, page 17).

b. While the rescue experiment in Figure 4C could provide some important insight into the down FMR1 using siRNA and verified that SG enrichment of hCdc73 was decreased (Fig. 4C). Then, we reconstituted cells with GFP-FMR1, which rescued the SG localization of hCdc73 (Fig. 4C, D)” A more precise description needs to be given (and included in the manuscript). Was the siRNA transfected on Day 1, the GFP-FMR1-encoding plasmid transfected 24 hours later, then the stress applied 24 hours afterward? Additionally, how did the plasmid derived FMR1 escape degradation by the siRNA? The siRNA target sequences correspond to amino acids 66-72 (siFMR1#1) and 98-

© 2023. Published by The Company of Biologists under the terms of the Creative Commons Attribution License (https://creativecommons.org/licenses/by/4.0/).

103 (siFMR1#2) of FMR1. These regions would also be present in the exogenous construct. Often, rescue experiments are performed using siRNA that targets the 5' or 3'-UTR of the mRNA (i.e., regions not found in the plasmid-derived transcript) or by mutating the siRNA binding site of the plasmid-derived FMR1 to avoid siRNA binding. More information is required to interpret these results.

□ To address the reviewer's comment, we added a more detailed description regarding the reconstitution procedure in the Materials and Methods section. (Materials and methods section, page 17).

□ To address concerns of reconstituted exogenous FMR1 degradation by siFMR#1 and siFMR#2, we performed a new experiment using FMR1 siRNAs that target the 3'-UTR (Supplementary table S1). We repeated the FMR1 reconstitution experiment with the new siRNAs, and the data are now presented in Figure 4C, D. We modified the manuscript accordingly (Results section, page 10).

c. Third, when analyzing the binding sites for FMR1 (above) this reviewer noted that these siRNAs appear to have been designed to target the mouse, not human FMR1 transcripts. In human HeLa cells, siFMR1#1 might be ok (one nucleotide doesn't align), but the efficiency of siFMR1#2 is extremely questionable (3 nucleotide mismatches). As this is a critical element of the manuscript, it warrants repeating with appropriately designed siRNA.

□ Based on the reviewer's comment, we redesigned siRNAs for FMR1 that target 5'-GGTGTATTCCAGAGCAAAT-3' of the coding region of FMR1. We also tested two different kinds of siRNAs targeting the 3'-UTR of FMR1, 5'-GTACTGAGCAGTGATATTC-3' and 5'-AAGTTGTATGATCTGTGCC-3'. We reperformed the FMR1 knockdown experiment with new siRNAs (Supplementary table S1), and the data are now presented in Figure 4A, B and 2) The authors have clarified the number of repeats for the statistical analysis ("We analyzed more than 10 cells by random selection among the three independent data sets"), but the graphs (Figure 2C, 2E, 4B, 4D, and 5I) are still confusing. The legend in Figure 2C/E states, "Quantification of … within SGs (n=11-12)" and there an 11-12 dots/squares/triangles for each sample. What do those dots mean? Are they the results form one cell in one repeat? Based on the authors description, shouldn't there be at least 30 dots (~10 for each of the three repeats)? Further clarification is required here.

□ We performed three biologically independent experiments. For each experiment, we randomly selected 3-4 cells for quantification. Hence, the total number of cells we quantitated ranged from 11-12.

□ While preparing the revision, we performed a new experiment and quantitated more cells to increase the total number of cells, as presented in Fig. 2 and Fig. 4. The new quantitation data are now presented in Fig. 2C, E and Fig. 4B, D. Additionally, to avoid confusion, we added the following sentences to the Materials section (Statistical analysis). "In the case of quantification of imaging data, at least 10 cells per experiment were randomly taken for quantitation. At least three biologically independent experiments are performed".

3) In Figures 4E, 4F, and S3D it appears that GST alone was added to some of the samples (e.g. lanes 1 and 3 of Figure 4E). This is not described in the figure or the figure legend and needs to be corrected.

□ For clarification, we placed positions of GST-alone, GST-hCdc73^{NLSmut}, and GST-tagged IDR mutants in the right panel in Figure 4E, 4F, S3D. Additionally, we added the following sentences to the figure legends: '-' indicates GST-empty or Flag-empty vector.

4) The authors have not actually addressed the comment, "The amino acid substitutions of the NLSmut construct contain typos in both Figure 1C and 2A. What does R126 and 139A (Figure 1C) and R126 and 13 (Figure 2A) mean?". Looking at the CDC73 sequence it appears that -7 Both 126 and 139 sites were changed from "R" to "A". We modified the figure accordingly.

© 2023. Published by The Company of Biologists under the terms of the Creative Commons Attribution License (https://creativecommons.org/licenses/by/4.0/).

Third decision letter

MS ID#: JOCES/2022/260593

MS TITLE: Translocation of cytosolic hCdc73 to stress granules plays a role in arsenic stress-induced stabilization of p53 mRNA

AUTHORS: Hojin Lee, Tae-Hyeon Kim, and Joo Yeon Yoo
ARTICLE TYPE: Research Article

I am happy to tell you that your manuscript has been accepted for publication in Journal of Cell Science, pending standard ethics checks.

© 2023. Published by The Company of Biologists under the terms of the Creative Commons Attribution License (https://creativecommons.org/licenses/by/4.0/).
